# Extravasation of Blood and Blood Toxicity Drives Tubular Injury from RBC Trapping in Ischemic AKI

**DOI:** 10.1093/function/zqad050

**Published:** 2023-09-04

**Authors:** Sarah R McLarnon, Chloe Johnson, Jingping Sun, Qingqing Wei, Gabor Csanyi, Phillip O'Herron, Brendan Marshall, Priya Giddens, Jennifer C Sullivan, Amanda Barrett, Paul M O'Connor

**Affiliations:** Department of Physiology, Medical College of Georgia, Augusta University, 30912, Augusta, GA, USA; Department of Cell Biology and Physiology, School of Medicine, University of North Carolina, 27599, Chapel Hill, NC, USA; Department of Physiology, Medical College of Georgia, Augusta University, 30912, Augusta, GA, USA; Department of Physiology, Medical College of Georgia, Augusta University, 30912, Augusta, GA, USA; Department of Anatomy and Cell Biology, Medical College of Georgia, Augusta University, 30912, Augusta, GA, USA; Department of Pharmacology and Toxicology, Augusta University, 30912, Augusta, GA, USA; Department of Physiology, Medical College of Georgia, Augusta University, 30912, Augusta, GA, USA; Department of Anatomy and Cell Biology, Medical College of Georgia, Augusta University, 30912, Augusta, GA, USA; Department of Physiology, Medical College of Georgia, Augusta University, 30912, Augusta, GA, USA; Department of Physiology, Medical College of Georgia, Augusta University, 30912, Augusta, GA, USA; Department of Pathology, Medical College of Georgia, Augusta University, 30912, Augusta, GA, USA; Department of Physiology, Medical College of Georgia, Augusta University, 30912, Augusta, GA, USA

**Keywords:** acute kidney injury, acute tubular necrosis, ischemia-reperfusion, renal

## Abstract

Red blood cell (RBC) trapping is common in ischemic acute kidney injury (AKI) and presents as densely packed RBCs that accumulate within and engorge the kidney medullary circulation. In this study, we tested the hypothesis that “RBC trapping directly promotes tubular injury independent of extending ischemia time.” Studies were performed on rats. Red blood cell congestion and tubular injury were compared between renal arterial clamping, venous clamping, and venous clamping of blood-free kidneys. Vessels were occluded for either 15 or 45 min with and without reperfusion. We found that RBC trapping in the medullary capillaries occurred rapidly following reperfusion from renal arterial clamping and that this was associated with extravasation of blood from congested vessels, uptake of blood proteins by the tubules, and marked tubular injury. To determine if this injury was due to blood toxicity or an extension of ischemia time, we compared renal venous and arterial clamping without reperfusion. Venous clamping resulted in RBC trapping and marked tubular injury within 45 min of ischemia. Conversely, despite the same ischemia time, RBC trapping and tubular injury were minimal following arterial clamping without reperfusion. Confirming the role of blood toward tubular injury, injury was markedly reduced in blood-free kidneys with venous clamping. Our data demonstrate that RBC trapping results in the rapid extravasation and uptake of blood components by tubular cells, causing toxic tubular injury. Tubular toxicity from extravasation of blood following RBC trapping appears to be a major component of tubular injury in ischemic AKI, which has not previously been recognized.

## Graphical Abstract

**Graphical Abstract:**
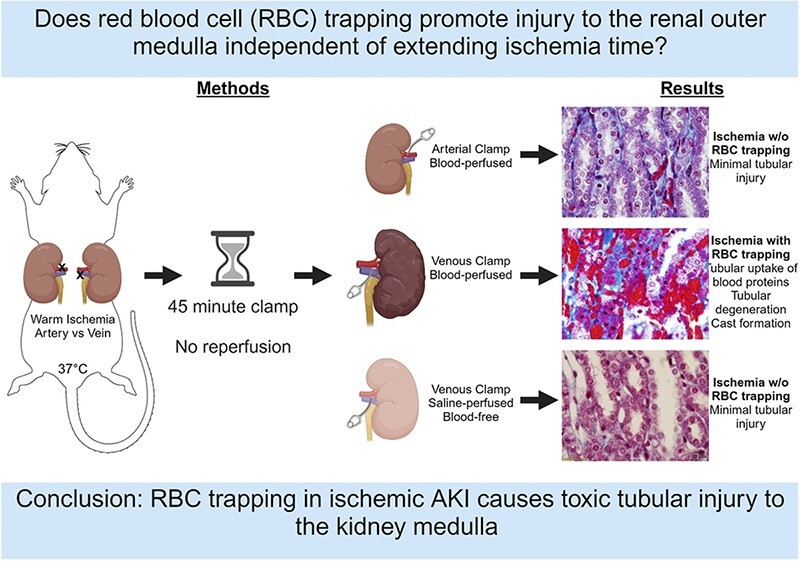

## Introduction

Red blood cell (RBC) trapping, often termed medullary vascular congestion or medullary hyperemia, presents as the formation of densely packed RBCs that accumulate within and engorge the renal outer-medullary (OM) circulation.^1,2^ Red blood cell trapping in the OM region of the kidney is a hallmark of ischemic acute kidney injury (AKI) with acute tubular injury (ATI) in humans.^2–8^ Red blood cell trapping is also present in animal models following ischemia reperfusion injury (IRI) from arterial clamping, including in pigs, dogs, rats, and mice,^9–12^ where it significantly contributes to the decline in kidney function.^1,13^

Our laboratory has recently reported that RBC trapping is strongly associated with severe tubular injury in the renal OM region and that this injury is evident in as little as 1 h following reperfusion following arterial clamping.^14^ It has been speculated that RBC trapping promotes kidney injury by delaying reperfusion and extending ischemic time to the RBC-congested areas of the kidney, thereby prolonging the period of tissue hypoxia. This period is commonly referred to as the extension phase of renal ischemia.^15,16^ Opposing this concept, however, blood flow is rapidly restored to the OM following reperfusion of the kidney,^14^ and there is little evidence of hypoxia in the renal OM region in the initial hours after experimental IR.^17,18^ Further, early tubular injury in congested areas of the kidney does not resemble typical ischemic/hypoxic injury, which is normally not evident in histological sections until 10-12 h post-ischemia.^19,20^

We and others have found that RBC trapping occurs following arterial IRI due to obstruction of the venous vessels that drain the renal medulla.^14^,^21–23^ The continued influx of blood into the medullary microcirculation while the venous vessels remain occluded, results in increased blood volume and vascular pressures within the medullary microcirculation. The increasing intravascular pressure then results in the extravasation of the plasma.^14,21,22,24^ Together, this explains the accumulation of ∼10 fold more RBCs in the OM microcirculation than are normally present, which expand the OM capillaries and form tightly packed aggregates with little or no plasma separating them.^2,14,21,23,25^

The goal of the current study was to determine whether RBC trapping directly causes toxic tubular injury independent of extending warm ischemia time. To do this, we compared kidney injury following a period of warm ischemia time without RBC trapping to kidney injury following the same warm ischemia time with RBC trapping. To promote the RBC trapping that normally occurs in the OM during the reperfusion phase during the earlier clamp phase, we utilized renal venous clamping. During renal venous clamping, the arterial vessels of the kidney remain unobstructed. As such, blood can enter the kidney, but it is unable to exit due to the venous clamp. This mimics what happens with the collapse of the venous vessels that drain the OM during reperfusion from arterial clamp ischemia.^14^ We hypothesized that this would result in RBC trapping during the ischemic period. Conversely, during the ischemic phase of arterial clamping, blood cannot enter the kidney, and RBC trapping is limited. Only when the arterial clamp is released does RBC significant trapping normally occur. As such, by comparing tubular injury between arterial and venous clamping without allowing reperfusion, we were able to examine the effects of RBC trapping on tubular injury while maintaining equal warm ischemia time. We hypothesized that RBC trapping during the ischemic phase would cause direct tubular injury.

## Methods

### Animals

All experiments were conducted in accordance with the National Institutes of Health “Guide for the Care and Use of Laboratory Animals” and were approved and monitored by the Augusta University Institutional Animal Care and Use Committee. Age matched male and female Wistar Kyoto (WKY) and Sprague–Dawley rats from Charles River Laboratories were used in all experiments. Initial studies were performed on WKY rats. As we were able to demonstrate similar responses in Sprague–Dawley rats, later studies were performed in these animals as they were less costly. Rats were housed in temperature (20-26°C) and humidity (30%-70%) controlled, 12:12 h light-cycled conventional animal quarters. Rats were provided ad libitum access to water and standard 18% protein rodent chow (Envigo Teklad, 2918).

### Warm Bilateral Ischemia-Reperfusion Surgery and Tissue Harvest

Ischemia-reperfusion was performed as previously described.^14,26,27^ Briefly, animals were anesthetized with ∼3% isoflurane and 95% oxygen. Body temperature (rectal probe) was maintained at ∼37°C for the duration of the surgery by a servo-controlled heating table and infrared heat lamp (R40, Satco S4998). The renal pedicles were accessed via left and right dorsal flank incisions. In some experiments, both renal arteries were clamped with Schwartz Micro Serrefines (Fine Science Tools #18052-03, Foster City, CA, USA) followed by 2 h of reperfusion. In other studies, the renal artery for one kidney and the renal vein for the other kidney were clamped for either 15 or 45 min without reperfusion. The kidney (left or right) that received arterial or venous clamping was alternated between animals. At the end of the clamp period, the kidneys were excised prior to the removal of the vascular clamp. The kidneys were fixed with VIP-Fixative (Fisher #23-730-587) or electron microscopy fixative (see below) for histological analysis. Animals that recovered from anesthesia were given buprenorphine for analgesia.

### Saline-Perfused, Blood-Free Perfusion Studies

Sprague–Dawley rats were prepared as above except, a midline incision was performed and the abdominal aorta cannulated. The mesenteric artery and vessels of the right kidney were ligated, and a loose tie placed around the abdominal aorta superior to the renal arteries. The renal vein was separated from the renal artery to allow clamp placement. The tie around the aorta was then retracted to prevent blood flow to the left kidney, and the kidney flushed of blood via retrograde perfusion of the abdominal aorta using warm ∼37°C saline. In blood-free animals, stable renal perfusion pressure at ∼100 mmgH was achieved using a purpose-made perfusion system consisting of a pressurized saline reservoir connected to a smaller in-line reservoir in which saline could be heated to 37°C before being administered. In animals in the blood-perfused group, perfusion pressure was maintained either at the level of arterial pressure (normal pressure group) or at less than 50 mmHg by adjusting a vascular occluder around the abdominal aorta to lower arterial perfusion pressure to the left kidney (low pressure group). Perfusion pressure to the renal artery was maintained across the 45-min venous clamp period. At the end of the 45-min clamp period, kidneys were immediately harvested and placed in a fixative solution, as per the first study.

### Assessment of RBC Congestion

Paraffin-embedded kidney sections were stained with Gomori’s trichrome (Thermo Scientific, Cat. No. 87020), according to the manufacturer’s instructions. Congestion of OM vasa recta (VR) and OM plexus capillaries was assessed using a semi-quantitative scoring method as previously described.^14^ Briefly, a score of 0-5 was given to reflect the extent of RBC congestion independently in each region. A score of 0 represents conditions in which all vessels appear open (0% congestion), and a score of 5 represents congestion in all vessels visualized (100% congestion).

### Assessment of Tubular Injury

Both trichrome and hematoxylin and eosin (H&E)-stained kidney sections were scored for tubular injury by a pathologist blinded to the hypothesis of the study and sample identifiers. Percent injury was scored for the entire cortex and medulla. Two to three sections from each animal were scored. For trichrome-stained sections, tubular injury (cell swelling/necrosis) and tubular cast formation were scored in both the cortex and OM. Tubular injury and cast formation are reported as a score of 0-5 with a score of 0 indicating 0%-5%, 1, indicating 5%-20%, 2, indicating 20%-40%, 3, indicating 40%-60%, 4, indicating 60%-80%, and 5, indicating 80%-100% of all tubules demonstrating that trait. For H&E-stained sections, 10-20 fields were scored covering the cortex and OM at magnification 20×, and the number of injured tubules divided by the total number of tubules scored and multiplied by 100.

### Immunostaining

Immunostaining was performed as previously described.^14^ For CD235a staining, slides were incubated with anti-CD 235a mouse monoclonal antibody (Invitrogen cat# MA5-12484) at 1:200 dilution in 10% goat serum in 0.1% phosphate-buffered saline with Tween (PBST) overnight at 4ºC. The next day, slides were washed 3 times in 0.1% PBST for 5 min each time before being incubated with a secondary antibody [goat anti-mouse IgG-HRP (Santa-Crus cat# sc-2005)] at 1:400 dilution in 10% goat serum in 0.1% PBST for 50 min at room temperature. Slides were then washed three times in 0.1% PBST for 5 min each time before chromogen staining with 3,3’-Diaminobenzidine (DAB) for 5 min. Slides were then rinsed 3 times in distilled water before being counterstained with hematoxylin for 1 min and washed in running tap water. Slides were covered using Cytoseal XYL medium (Thermo Scientific cat# 8312-4). Hemoglobin (Hb) staining was performed as per CD235a except, Hb alpha recombinant rabbit monoclonal antibody from ThermoFisher, cat# MA5-32328, 1 mg/mL was used at a dilution of 1:200. No primary antibody was used as a control (Supplementary Figure S1).

### Western Blots

To examine the specificity of anti-Hb and anti-CD235a antibodies, 3 male WKY were utilized for RBC and kidney cortex tubule protein extraction. For the collection of RBC, rats were anesthetized with isoflurane (3%-5%), and blood was collected by cardiac puncture using a heparinized syringe. Blood was then spun at 400 g, and the pellet collected and re-suspended in saline. This was repeated three times and the remaining RBC pellet was used for membrane protein isolation. For proximal tubular cells, the left and right kidneys were perfused with ∼10 mL 200 unit/mL collagenase IV in HEPES buffered Hanks’ Balanced Salt Solution (HBSS) pH 7.4. The kidneys were then excised and the cortex isolated by dissection and cut into small pieces by a razor blade. Cortical pieces were then placed into the same collagenase solution and incubated at 37°C for 4 min. The supernatant was then collected into a glass pipette and filtered consecutively through 250 µm and then 75 µm metal sieves. The 75-µm sieve was used to collect proximal tubule fragments. Once proximal tubule fragments collected, the 75-µm sieve was flipped and the underside washed using ice chilled 1% bovine serum albumin in HBSS solution pH 7.4 to stop the collagenase digestion and collect the tubules. The resulting fluid was collected into a 50-mL conical tube and spun at 200 g for 5 min at 4°C. The resulting pellet was confirmed to contain primarily tubular fragments by inspection under a light-microscope. The pellet was then washed in 15 mL of HBSS before being spun at 200 g for 5 min at 4°C, and the final pellet collected. We used the Mem-Per Plus Membrane Protein Extraction Kit (Thermo Scientific cat# 89842) for membrane protein and cytoslic protein extraction following the manufacturer’s instructions. Protein concentration was determined (Bradford cat# 5000205, BioRad protein assay), and 50 µg RBC and kidney cortex tubule membrane protein was loaded per lane for the CD235a blot. A concentration of 30 µg of RBC and proximal tubular cytosolic protein was loaded per lane for the Hb blot. Protein was loaded onto 4%–20% mini-protein TGX gel for electrophoresis before transfer to a Immobilon-FL membrane (Millipore Cat# IPFL00010). The blot was first incubated with a primary antibody (CD235a 1:100, Hb 1:1500, overnight at 4°C), followed by a secondary antibody (goat anti-mouse IRDye 680RD, Invitrogen cat# 926-68070, 1:5000 for CD235a, goat anti-rabbit IRdye 680RD, Invitrogen cat# 926-68071, 1:5000 for Hb). The blot was scanned by a Li-Cor Odyssey CLx. The blot was then washed with 1xTBS before sequential incubation with anti-B-actin rabbit antibody for Hb cytosolic protein. As RBC did not show a band for β-actin, we used Ponceau staining as our loading control. We use 0.1% Ponceau Red dye 5% glacial acetic acid to stain our polyvinylidene difluoride membranes saturated in ∼15 min, then rinsed with distilled H_2_O before capturing images (Supplementary Figures S1 and S2).

### Evans Blue Imaging

Evans blue (EB) binds strongly to plasma proteins creating a large molecular weight tracer that can been used to investigate plasma extravasation in tissues.^24,28,29^ To confirm the extravasation of blood proteins, we injected 50 mg/kg of EB in 1 mL of saline into the tail vein of rats 20 min prior to arterial (*n* = 3) or venous clamping (*n* = 3) without reperfusion. Following 45 min of clamp ischemia, kidneys were harvested without removing the clamps, bisected and placed in a fixative solution. In addition, we also compared EB staining between control rats (*n* = 3, no IRI) and rats following 2 h of reperfusion from 45 min of clamp ischemia. In these animals, EB was injected into the tail vein during the clamp period and allowed to circulate until tissue harvest. A concentration of 5-µm thick, unstained paraffin-embedded kidney sections were imaged. Slides were imaged using an Olympus FLUOVIEW™ FV3000 Confocal Laser Scanning Microscope using a 40× oil objective at 1024 × 1024 resolution. A 488 nm laser (0.6% power) with emission detected between 500 and 540 nm was used to image green autofluorescence from the kidney. Evans blue was imaged using a 640 nm laser for excitation (0.07%) with emission light detected between 620 and 720 nm. The same microscope settings were used for all images.

### Endogenous DAB Staining

3,3’-DAB staining was used to visualize the endogenous peroxidase activity of Hb.^30–32^ Four male WKY rats were used for DAB staining. A 45-min warm unilateral arterial clamp ischemia was performed followed by 2 h of reperfusion before harvesting the right (ischemic) and left (control) kidneys and placing in fixative solution. Paraffin-embedded sections of both control and IR tissue were cut at 5 µm and mounted on the same slide. Slides were then deparaffinized and hydrated to deionized water. Re-hydrated sections were then placed in a dark chamber and incubated with DAB solution (Biocare Medical Betazoid DAB Kit cat# BDB2004L) for 20 min, followed by rinsing in distilled water. Stained sections were counterstained with hematoxylin for 1 min, dehydrated in an ethanol series, and cleared in xylene before mounting. *N* = 3 slides each from rats undergoing either 45 min of either arterial or venous clamping without reperfusion or 45 min of venous clamping with saline perfusion were also stained for DAB.

### Prussian Blue Staining

Sections from the same animals stained for DAB following reperfusion from arterial clamping were also stained with Prussian blue to detect ferrous, non-heme bound iron. ABCAM Iron Stain Kit (Prussian Blue Stain, cat# ab150674) was used for Prussian Blue staining per the manufacturer’s instructions. In brief, the slides were deparaffinized and hydrated in distilled water. Equal volumes of potassium ferrocyanide solution and hydrochloric acid solution were mixed to make the iron stain solution. Slides were then incubated with the iron staining solution for 3 min before being rinsed slide in distilled water. The slides were then counterstained with a nuclear fast red solution for 5 min. Slides were then rinsed in 4 changes of distilled water, dehydrated, and cleared before mounting.

### Transcutaneous Clearance Measurements

Glomerular filtration rate (GFR) was determined using transcutaneous FITC-Sinistrin clearance^33^ in 11 male WKY rats. Transdermal mini-GFR monitors and measurement software (Medibeacon Inc., St. Louis, MO, USA) were used to determine GFR over a 2-h window of time. Rats were anesthetized with isoflurane (2%-5%), and transdermal mini-GFR monitors were placed between the shoulder blades using a double-sided adhesive patch. A volume 20 mg/kg of FITC sinistrin was then injected into the tail vein. The ½ life of FITC-Sinistrin clearance was determined using the MBLAB software [(Medibeacon Inc., St. Louis, MO, USA] and converted to GFR in mL/min using a 1-compartment model, according to the manufacturer’s instructions. Glomerular filtration rate was determined this way in 6 control rats without ischemia surgery and in 5 rats 0-2 h and ∼24-26 h (4 of 5) into reperfusion following 45 min of warm bilateral arterial clamping. At the time of harvest, thin (1-2 mm) sections of the kidney were cut and visualized on a fluorescent microscope (Ex500nm and Em515nm) to visualize FITC sinistrin.

### Transmission Electron Microscopy

Preparation and imaging of kidney tissue were performed by the Augusta University Histology Core Facility. Tissue was fixed in 4% paraformaldehyde and 2% glutaraldehyde in 0.1 m sodium cacodylate (NaCac) buffer, pH 7.4, postfixed in 2% osmium tetroxide in NaCac, stained en bloc with 2% uranyl acetate, dehydrated with a graded ethanol series, and embedded in Epon-Araldite resin. Thin 75-nm sections were cut with a diamond knife on a Leica EM UC6 ultramicrotome (Leica Microsystems, Bannockburn, IL, USA), collected on copper grids, and stained with uranyl acetate and lead citrate. Tissue was observed in a JEM 1400 Flash transmission electron microscope (JEOL USA, Peabody, MA, USA) at 120 kV and imaged with a Gatan 1095 OneView camera (Gatan, Pleasanton, CA, USA).

### Statistics

Graphpad Prizm was utilized for all data analysis. The *n* numbers and the specific statistical test utilized for individual experiments are listed in each figure legend. No data were excluded for any reason other than a failed experiment, the criteria for which was pre-defined prior to analyzing the data.

## Results

**Figure 1.**
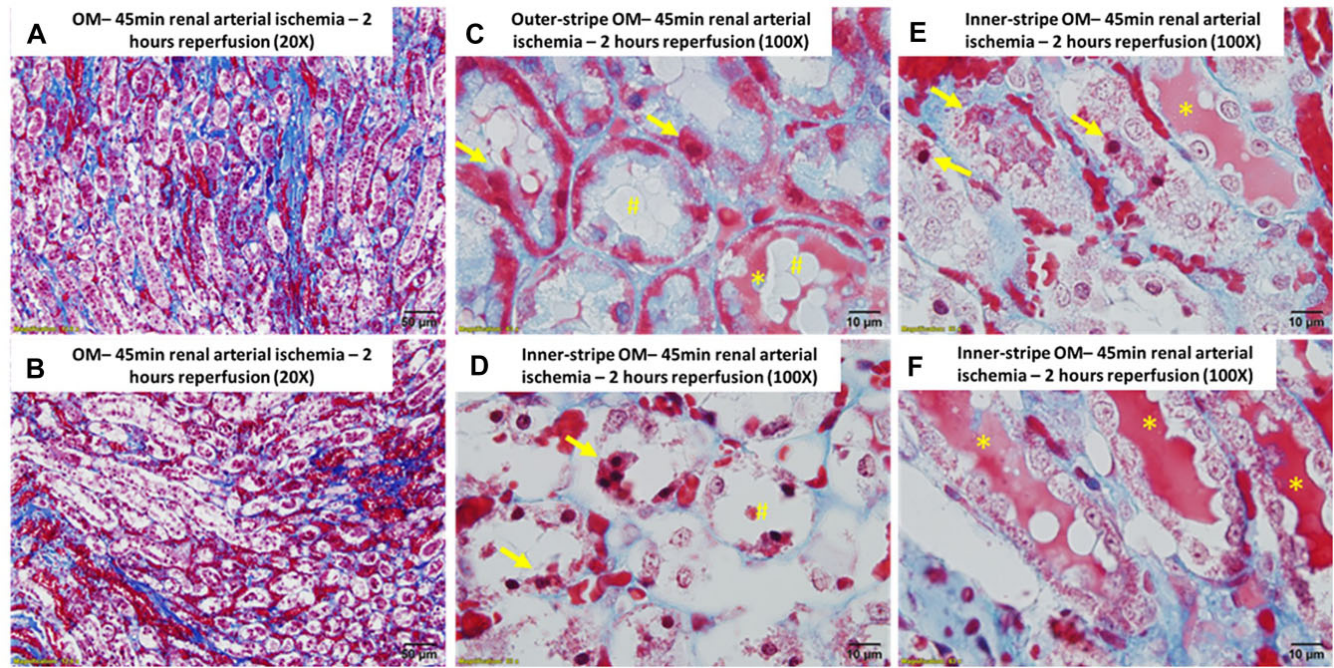
Arterial clamping with reperfusion: Representative images of trichrome-stained sections of the rat outer-medulla 2 h following reperfusion from 45 min of warm, bilateral, and arterial clamp ischemia. (Panel A), 20× image of the outer-medulla (OM). There is significant RBC vascular congestion (dark red). Almost all tubule cells in the inner stripe of the OM appear detached from the basement membrane. (Panel B), 20× image of the OM of another rat. There is marked RBC congestion of the VR and peritubular capillaries with RBCs. Almost all tubule cells in the congested inner stripe of the OM have sloughed into the lumen. (Panel C), 100× image of the outer stripe of the outer-medulla. Many tubular cells and their nuclei appear bright red. (arrows) There is prominent blebbing (#) and red-cast material in the tubular lumen (*). (Panel D), 100× image of the inner stripe of OM of the rat shown in Panel B. Almost all tubules appear to have degenerated and have large numbers of cells sloughed into the tubular lumen (#). Injured tubular cells take on a bright red appearance with darkened nuclei (arrows). (Panel E), 100× image of the inner stripe of OM of the rat shown in Panel A. Many tubular cells appear swollen with blebbing and intraluminal casts (*). Note the red droplets within and dark red nuclei of some cells (arrows) compared with surrounding cells. (Panel F), 100× image of inner stripe of OM. Note prominent intraluminal casts (*), cell blebbing, and swelling. The kidneys of 3 rats were examined, all demonstrating similar morphology. Scale bars are shown in each image.

We first examined whether severe OM tubular injury following 2 h of reperfusion from renal arterial clamping and the development of RBC trapping in the OM is associated with extravasation and tubular uptake of blood. Trichrome-stained sections of rat kidneys following 2 h of reperfusion from warm bilateral arterial clamping confirmed severe tubular injury in the RBC-congested OM, with almost all tubular cells having sloughed from the basement membrane (Figure 1A and B). Many of these injured tubular cells appeared to turn red or contain red droplets that were largely indistinguishable in color from RBCs (Figure 1C and D). Tubular degeneration and luminal cast formation were also prominent (Figure 1E and F). Vasa recta contained congested RBCs and pigmented material (Figure 1A and B).

**Figure 2.**
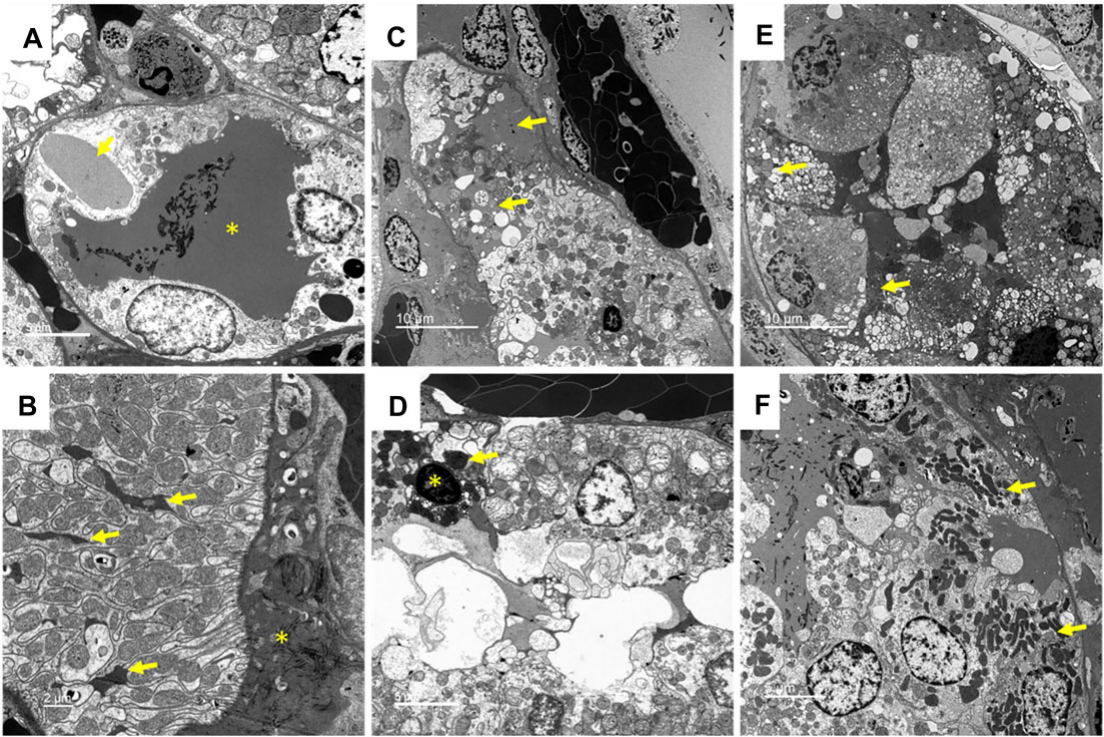
Arterial clamping with reperfusion: Representative, high-power transmission electron microscopy images of the rat outer-medulla 2 h following reperfusion from 45 min of warm, bilateral, and arterial clamp ischemia. (Panel A), the lumen of many tubular cells contains electron-dense material (*). Large electron-dense droplets of varying density can be found within tubular cells (arrows). (Panel B), Electron-dense material (*) is found in the interstitial space between RBC-congested capillaries and tubular cells. Often the basolateral membrane invaginations are darkened, and electron-dense material can be seen to accumulate within these channels (arrows). (Panel C), The cytosol of tubular cells surrounded by electron-dense material is often darkened (arrows). Note the much lower density of the cytosol of nearby tubular cells. (Panel D), Often, mitochondria (arrow) and nuclei (*) were found to be darkened in cells with electron-dense material within basolateral invaginations. Note the much lower density of mitochondria and nucleus of adjacent cells. Significant cell sloughing/blebbing into the lumen is also observed in this image. (Panel E), Tubular injury is prominent. Note the cellular sloughing/blebbing into the lumen filled with electron-dense material. Darkened cell cytosol and cellular vacuolization (arrows). (Panel F), Electron-dense material appears continuous between vessel, interstitial space, and the basolateral side of tubular cell sloughing of basement membrane. Tubular cells contain darkened mitochondria (arrows). Tubular lumen is filled with electron-dense material.

Electron microscopy confirmed detachment of tubular cells from the basolateral membrane, cellular vacuolization, and membrane blebbing (Figure 2). Many cells contained large electron-dense droplets of various density (Figure 2A). Often, the basolateral invaginations of tubular cells appeared engorged with electron-dense material (Figure 2B). The cytosol of many cells was darkened (Figure 2C). Many cells also contained electron-dense intraluminal casts (Figure 2A and E). In a number of cells, the mitochondria or nuclei were darkened, similar to that reported by Janike following cell-free Hb toxicity (Figure 2D and F).^34^

**Figure 3.**
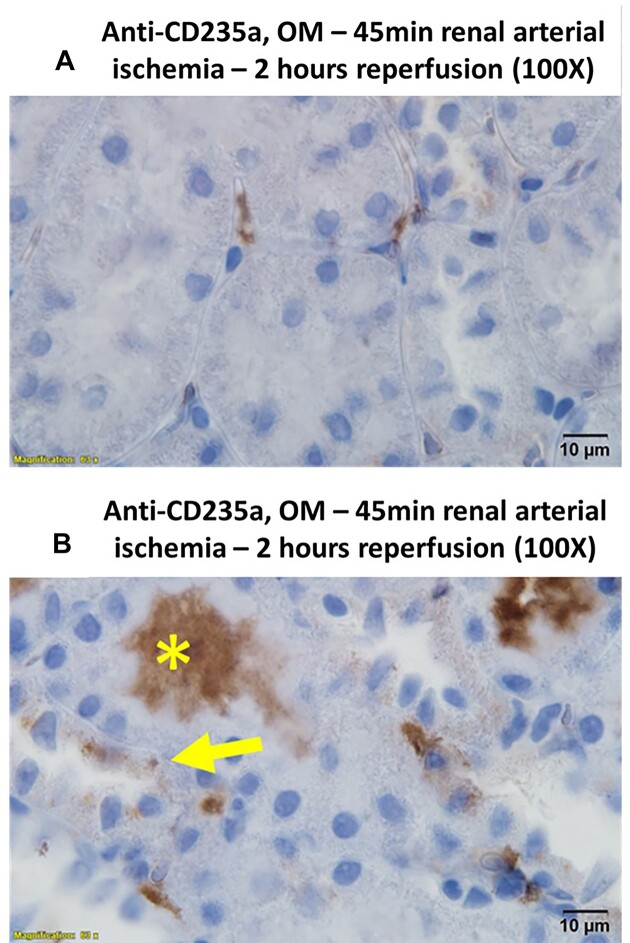
Arterial clamping with reperfusion: RBC membrane protein CD235a is concentrated in tubular casts and tubular cells in the outer-medulla following 45 min of arterial clamping with 2 h of reperfusion. Representative 100× images are shown of the cortex (Panel A) and outer-medulla (OM) (Panel B) of rats following 2 h of reperfusion from 45 min of warm, bilateral, and arterial clamp ischemia. CD235a is shown in brown. Tissue is counterstained with hematoxylin. Note the luminal cast material stains positive for CD235a (Panel B, *). Tubular cells in the OM also stain diffusely positive for CD235a (Panel B, arrow). In contrast, tubular cells in the renal cortex are mostly negative for CD245a staining. Only mild CD235a staining is observed within RBCs contained within capillaries (Panel A).

**Figure 4.**
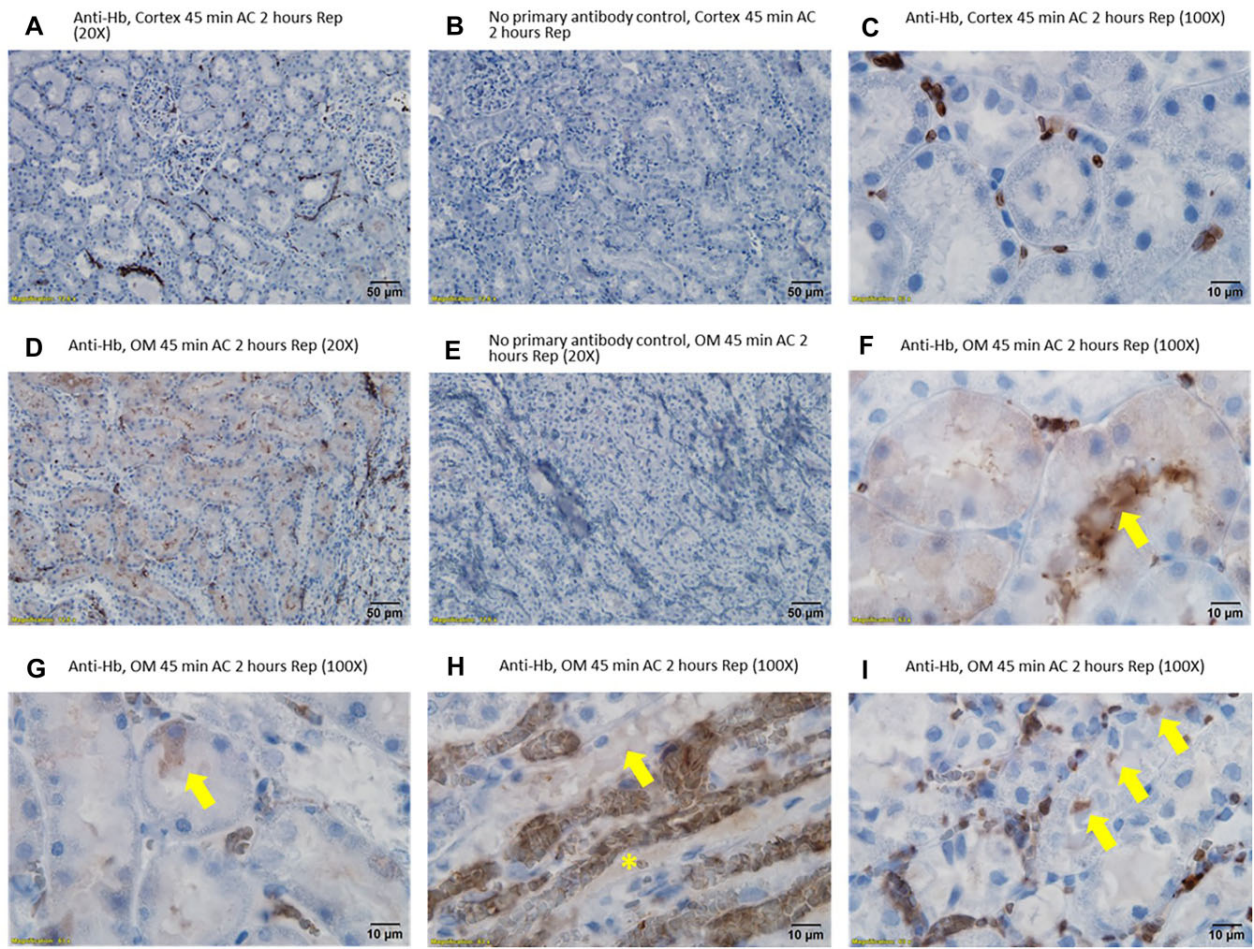
Arterial clamping with reperfusion: Hb is found in tubular cells and tubular casts of outer-medulla following 2 h of reperfusion from arterial clamping. (Panel A), low-power (20× original magnification) representative image of the rat kidney cortex 2 h after reperfusion from 45 min of warm bilateral arterial clamping (AC). Only RBCs in the vasculature stain positive for Hb. (Panel B), No-primary antibody control section of the kidney cortex. Positive staining is absent. (Panel C), high-power (100× original magnification) representative image of the rat kidney cortex 2 h after reperfusion from 45 min of warm bilateral AC. Only RBCs in the vasculature stain positive for Hb. Tubular cells are negative. (Panel D), low-power (20× original magnification) representative image of the rat kidney outer medulla (OM) 2 h after reperfusion from 45 min of warm bilateral AC. Red blood cells in the vasculature stain positive for Hb. Tubular cells in the OM and luminal casts also stain positive for Hb. (Panel E), No-primary antibody control section of kidney OM. Positive staining is absent. (Panel F), High-power (100× original magnification) representative image of the rat kidney OM 2 h after reperfusion from 45 min of warm bilateral arterial clamping. Red blood cells in the vasculature stain positive for Hb. Many tubular cells are lightly positive. Luminal casts are also positive for Hb (arrow). (Panel G), Same as for Panel F. In this example, a single tubular cell in a tubular cross section stains positive for Hb (arrow). (Panel H), High-power (100× original magnification) representative image of the inner stripe of the rat kidney OM 2 h after reperfusion from 45 min of warm bilateral arterial clamping. Red blood cells in the congested VR stain positive for Hb. There is evidence of free Hb in the VR without RBCs (*). Tubular cell staining is observed (arrow). (Panel I), Same as for Panel H. In this example, sloughed tubular cells stain positive for Hb (arrows).

**Figure 5.**
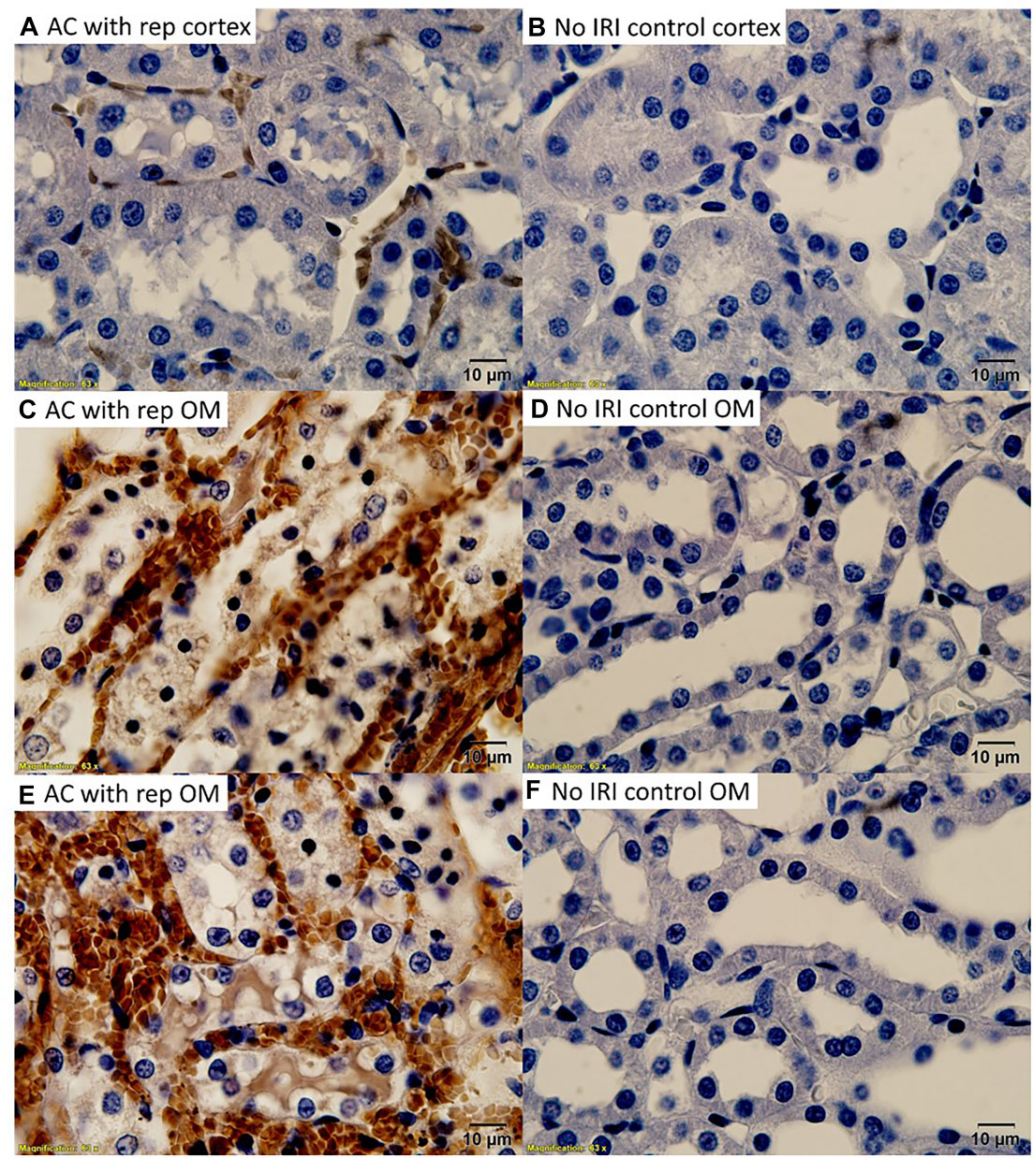
Arterial clamping with reperfusion and no ischemia control kidneys: DAB staining for endogenous Hb peroxidase activity. Representative images of 3,3’-DAB staining for endogenous peroxidase activity from male WKY rats. Images in the left panels (Panels A, C, and E) are from kidney that underwent 45 min of arterial clamp ischemia with 2 h of reperfusion (AC with Rep). Images in the right panels (Panels B, D, and F) are from control kidneys from the same animal that did not undergo ischemia reperfusion (no IRI control). Images are representative of *n* = 4 animals observed. Tubular DAB staining is minimal in the cortex, with only RBCs within the vasculature staining positive (Panel A). Red blood cells stain positive due to the endogenous peroxidase activity of Hb. In the outer-medulla (OM), injured tubular cells contain diffuse DAB staining (Panels C and E). Tubular casts also stain positive for DAB (Panels C and E). In controls, tubular DAB staining is absent in the cortex (Panel B) and OM (Panels D and E). All sections were stained at the same time, and sections from both ischemic and control kidneys from the same animal were mounted and stained on the same slide. Original magnification 100×.

The identity of the electron-dense material within tubular cells and intraluminal casts was further investigated by immunohistochemistry. CD235a, or glycophorin A, is a protein that is highly expressed in the cell membrane of erythrocytes.^35^ CD235a staining localized primarily to blood vessels; however, droplets within tubular cells and the electron-dense tubular casts in the OM also stained strongly positive (Figure 3). Some cortical tubules demonstrated CD235a-positive droplets; however, this was uncommon (Figure 3). Hemoglobin staining followed a similar pattern with both the tubular casts and injured tubular cells of the OM staining positive (Figure 4). Western blot confirmed the specificity of the anti-Hb antibody, demonstrating a single band at the correct molecular weight in homogenates of RBCs and no significant signal from saline-flushed whole kidney homogenates (Supplementary Figure S1). Western blot analysis of the CD235a antibody revealed a single band at the correct molecular weight for CD235a in membrane fractions from RBCs. However, a second band was also observed in membrane fractions from isolated proximal tubular cells (Supplementary Figure S2). The localization of Hb staining was confirmed using 3,3'-DAB. Hemoglobin acts as an endogenous peroxidase. Oxidized DAB resulted in the deposition of a brown precipitate that localized to RBC and OM tubules in areas of RBC trapping^30–32^ (Figure 5). Consistent with both anti-CD235a and Hb staining, the cortical tubules were mostly negative for DAB staining (Figure 5A). Both the tubular cytosol of OM tubules and tubular casts stained positive for endogenous Hb peroxidase activity using DAB (Figure 5C and E).

**Figure 6.**
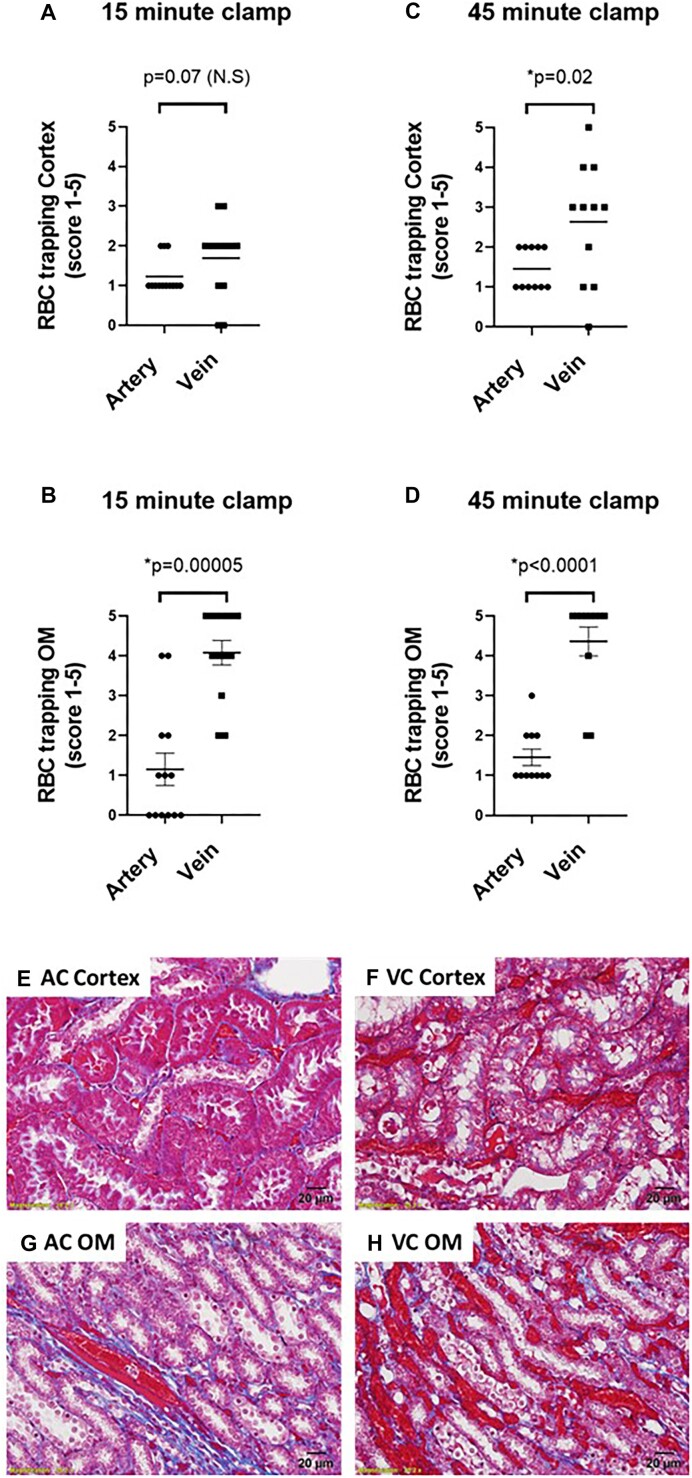
Arterial and venous clamping without reperfusion: The effect of arterial versus venous clamping on RBC trapping in the cortex and outer-medulla. Red blood cell trapping scores of the cortex or outer-medullary (OM) capillary plexus following 15 (Panel A and B, respectively) or 45 min (Panel C and D, respectively) occlusion of the renal artery (AC) or renal vein (VC) with no reperfusion in male (*n* = 7) and female (*n* = 6) WKY rats. Vascular congestion scores: 0 = 0%, 1 = 20%, 2 = 40%, 3 = 60%, 4 = 80%, and 5 = 100%. Values are expressed as mean ± SEM. Mann–Whitney test was used to compare clamp position, *P < 0.05. Note: for each rat, the renal vein was clamped on one kidney and the renal artery was clamped on the other kidney. (Panel E), Representative images of trichrome-stained sections of the cortex following 45-min occlusion of the renal artery with no reperfusion. There is only moderate congestion of the cortical capillaries. (Panel F), Representative images of trichrome-stained sections of the cortex following a 45-min occlusion of the renal vein with no reperfusion. Cortical capillaries are congested with RBCs (dark red between tubules). (Panel G), Representative images of trichrome-stained sections of the OM following a 45-min occlusion of the renal artery with no reperfusion. Only the VR bundles appear congested. (Panel H), Representative images of trichrome-stained sections of the OM following a 45-min occlusion of the renal vein with no reperfusion. Vasa recta bundles and capillary plexus are packed with RBCs. Images were taken at 40×. Scale bar denotes 20 µm.

To determine the direct effect of RBC trapping on tubular injury, we needed a model in which we could promote RBC trapping prior to the reperfusion phase during the clamp period. As such, we tested the hypothesis that “renal venous clamping results in greater OM RBC trapping during ischemia than arterial clamping.” Moderate RBC trapping was present in the cortical peritubular capillaries and veins following both 15 and 45 min of ischemia from either renal arterial or venous clamping without reperfusion. However, RBC trapping was significantly greater following 45 min of venous clamping compared with 45 min of arterial clamping (Figure 6). In the OM capillary plexus, renal venous occlusion resulted in significantly greater RBC trapping than arterial clamping at both 15 (Figure 6B, *P* < 0.0001) and 45 min (Figure 6D, *P* < 0.0001) of clamping. The larger VR vessels were congested following both arterial and venous clamping; however, the VR were more engorged with RBCs following venous clamping (Figure 6G and H).

**Figure 7.**
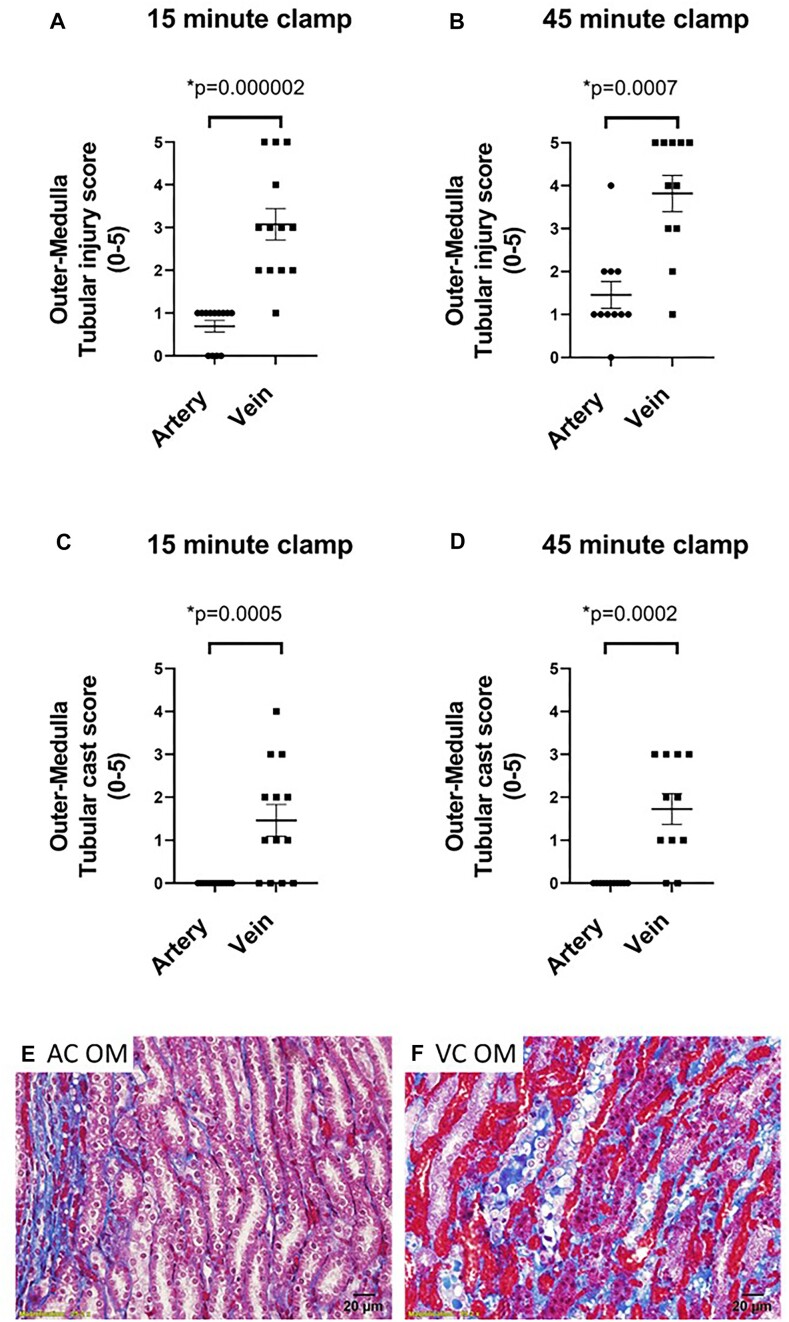
Arterial and venous clamping without reperfusion: The effect of arterial versus venous clamping on outer-medullary tubular injury. Tubular injury scores of the outer medulla (OM) following 15 (Panel A) or 45 min (Panel B), of occlusion of the renal artery (AC) or renal vein (VC) with no reperfusion in male (*n* = 7) and female (*n* = 6) WKY rats. Tubular cast formation scores of OM following 15 (Panel C) or 45 min (Panel D) occlusion of the renal artery or renal vein with no reperfusion. Tubular injury or cast formation is reported as 0-5, representing the % of tubules demonstrating tubular injury with a score of 0 indicating 0%-5%, 1, indicating 5%-20%, 2, indicating 20%-40%, 3, indicating 40%-60%, 4, indicating 60%-80%, and 5, indicating 80%-100% of all tubules demonstrating that trait. Values are expressed as mean ± SEM. Mann–Whitney test was used to compare clamp position, *P < 0.05. Note: for each rat, the renal vein was clamped on one kidney and the renal artery was clamped on the other kidney. (Panel E), Representative image of tubular morphology in trichrome-stained sections of the OM following 45 min of occlusion of the renal artery with no reperfusion. Tubular morphology remains relatively normal following arterial clamping. (Panel F), Representative image of tubular morphology in trichrome-stained sections of the OM following 45 min of occlusion of the renal vein with no reperfusion. Swollen pale cells are common, often with blue or red casts within their lumen. Following 45 min of venous clamping, tubular necrosis is also observed, as evidenced by darkened pyknotic nuclei, loss of cellular cytoplasmic area, and tubular structure. Images taken at 40×.

As renal venous clamping promoted significant RBC trapping during the clamp period, we next tested the hypothesis that “tubular injury would be greater following renal venous clamping without reperfusion, compared with renal arterial clamping without reperfusion, independent of warm ischemia time.” Following 15 min of renal venous clamping without reperfusion, there was significant tubular injury in the renal OM. This included cell swelling and tubular cast formation. In contrast, few tubules in the OM region demonstrated significant tubular injury following 15 min of arterial clamping without reperfusion (Figure 7A, *P* < 0.0001). When ischemic time was increased to 45 min, tubular injury following venous clamping without reperfusion increased. This included evidence of pyknotic nuclei, loss of tubular structure, cellular swelling, and cast formation (Figure 7B and F). In contrast, the percentage of injured tubular cells remained lower, and tubular morphology looked relatively normal following 45 min of arterial clamping without reperfusion (Figure 7B and E).

**Figure 8.**
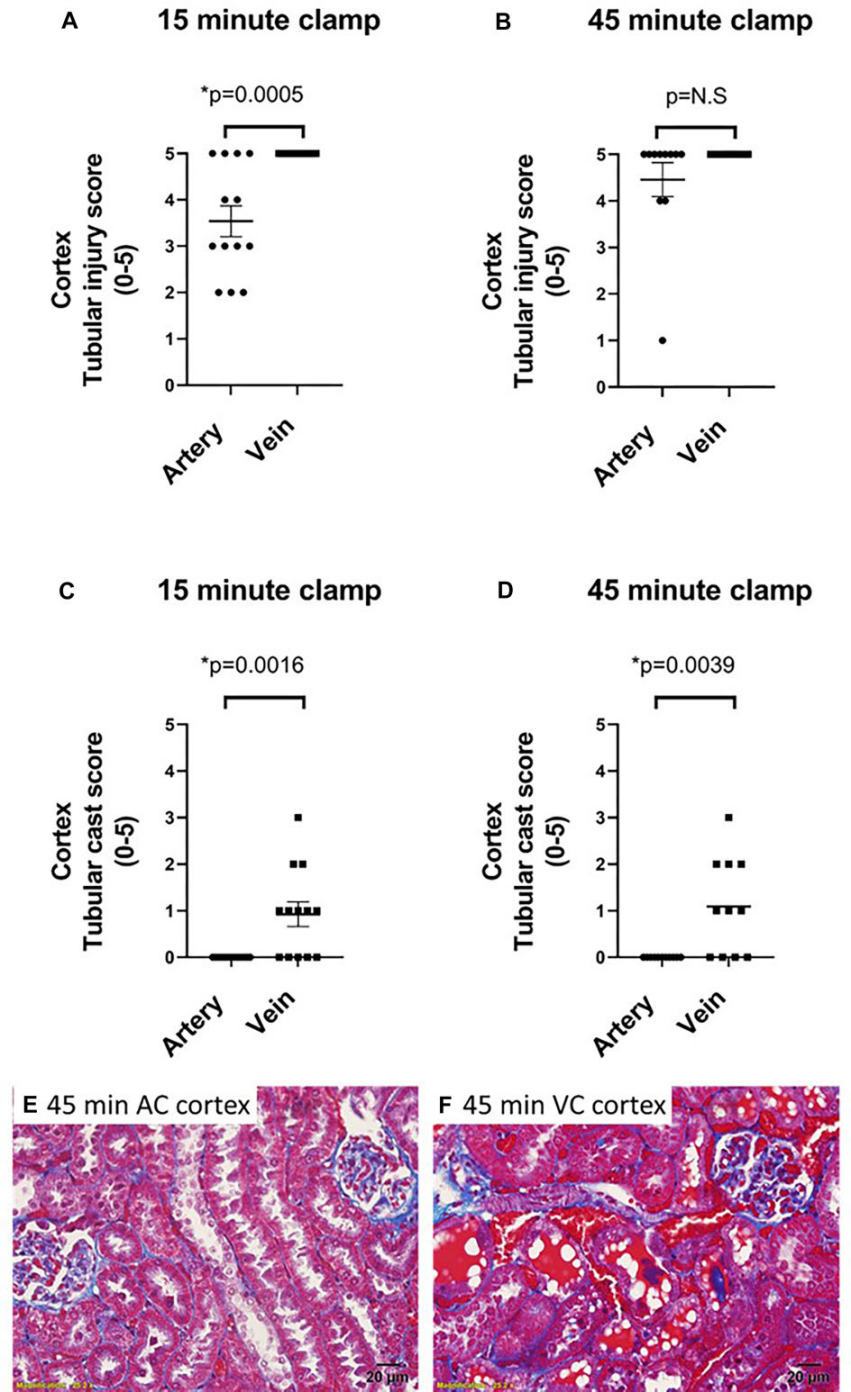
Arterial and venous clamping without reperfusion: The effect of arterial versus venous clamping on cortical tubular injury. Tubular injury scores of the kidney cortex following 15 (Panel A) or 45 min (Panel B) of occlusion of the renal artery (AC) or renal vein (VC) with no reperfusion in male (*n* = 7) and female (*n* = 6) WKY rats. Tubular cast formation scores of the cortex following 15 (Panel C) or 45 min (Panel D), occlusion of the renal artery or renal vein with no reperfusion. Tubular injury or cast formation is reported as 0-5, representing the % of tubules demonstrating injury with a score of 0 indicating 0%-5%, 1, indicating 5%-20%, 2, indicating 20%-40%, 3, indicating 40%-60% 4, indicating 60%-80%, and 5, indicating 80%-100% of all tubules demonstrating that trait. Values are expressed as mean ± SEM. Two-way ANOVA comparing clamp position and sex, *P < 0.05. Note: for each rat, the renal vein was clamped on one kidney and the renal artery was clamped on the other kidney. (Panel E), representative trichrome-stained 20× image of the kidney cortex following 45 min of arterial clamping without reperfusion. (Panel F), representative trichrome-stained 20× image of the kidney cortex following 45 min of venous clamping without reperfusion. Casts were almost exclusive to the venous clamped kidney. Following 15 min of venous clamping, casts often appeared as discrete droplets within the tubular lumen rather than the continuous casts filling the entire lumen observed at 45 min of clamping (Figure 4F). Images were taken at 40×. Casts appeared more developed following 45 min of venous clamping when compared with 15 min of clamping.

**Figure 9.**
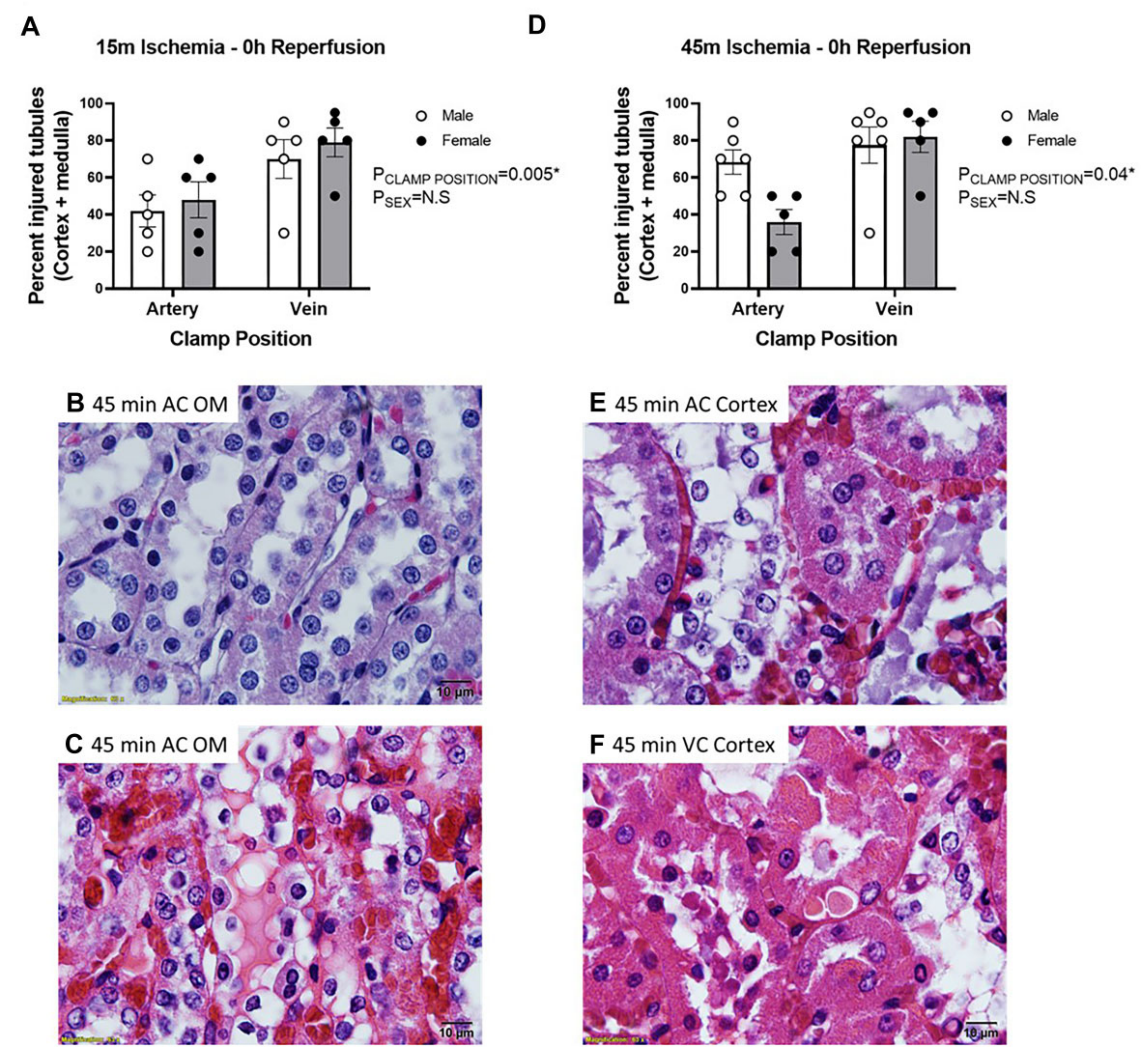
Arterial and venous clamping without reperfusion: H&E injury scoring. Injury was scored using H&E-stained slides as % of injured tubules [aggregate score of cortex and outer-medulla (OM)] by a researcher unaware of the hypothesis being tested or the sample identifiers. (For Panels A and D), *Y*-axis, % of tubules in the cortex and OM demonstrating signs of injury; *X*-axis, clamp position; males are shown as open circles; females are shown as closed circles. Data are mean ± SEM. Statistics are a result of a 2-way ANOVA comparing the effect of clamp position and sex. (Panel A), Aggregate tubular injury score (% of tubules with injury) in cortex and outer-medulla (OM) following 15 min of arterial (AC) or venous (VC) clamping without reperfusion. (Panel D), Aggregate tubular injury score (% of tubules with injury) in cortex and OM following 45 min of arterial or venous clamping without reperfusion. (Panels B, C, E, and F), Representative 100× images of the inner stripe of the outer-medulla and cortex of H&E-stained kidney sections. (Panel B), representative image of OM of rat following 45 min of arterial clamping without reperfusion. Red blood cell congestion of the plexus vasculature is minimal, and tubular architecture remains relatively normal. (Panel C), representative image of OM of rat following 45 min of venous clamping without reperfusion. The plexus vasculature is congested with RBCs (dark red between tubules). Tubular architecture is disrupted. There is cell swelling and blebbing. Casts are observed in the tubular lumen. (Panel E), representative image of the cortex of rat following 45 min of arterial clamping without reperfusion. Much of the peritubular vasculature is congested with RBCs. Tubular architecture is disrupted. Some tubular cells are swollen. There is blebbing/sloughing of tubular cell material into the tubular lumen. (Panel F), representative image of the cortex of rat following 45 min of venous clamping without reperfusion. Similar to the cortex following arterial clamping, much of the peritubular vasculature is congested with RBC. Tubular architecture is disrupted. Some tubular cells are swollen. There is blebbing/sloughing of tubular cell material into the tubular lumen.

There was also evidence of tubular injury in the kidney cortex. Following 15 min of venous or arterial clamping without reperfusion cortical injury was greater with renal venous compared with renal arterial clamping (Figure 8A, *P* = 0.003). Tubular cast formation in the renal cortex was exclusive to animals in which the renal vein was clamped (Figure 8C–F). Tubular injury scores were similar when injury was assessed using H&E-stained sections (Figure 9).

**Figure 10.**
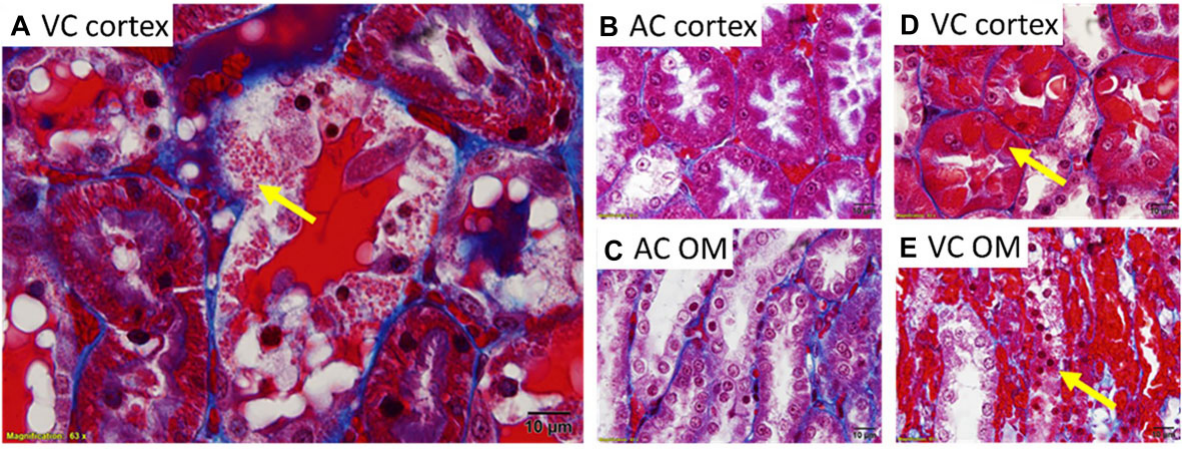
Arterial and venous clamping without reperfusion: The effect of arterial versus venous clamping on tubular appearance. (Panel A), Representative image of a trichrome-stained section of the kidney cortex following 45 min of renal venous clamping (VC). Red droplets (heme loaded mitochondria?) can be observed to have accumulated within the tubular structures, causing them to appear bright red in trichrome-stained sections (arrow). (Panel B), Representative image of a trichrome-stained section of the cortex following a 45-min renal artery clamping (AC) with no reperfusion. Some red droplets are observed in cortical tubules; however the density of these is much less than that following venous clamping. (Panel C), Representative image of a trichrome-stained section of the kidney outer-medulla (OM) following 45 min of arterial occlusion. Tubular cells have little to no evidence of RBC uptake. (Panel D), Representative image of a trichrome-stained section of the cortex following 45-min occlusion of the renal vein with no reperfusion. Tubular cells become deeply red (arrow) and red casts can be observed in the lumen. Note the color change in the cytosol of tubular cells when comparing 45 min of arterial or venous clamping as in Panels B and D. (Panel E), Representative image of a trichrome-stained section of the kidney OM following 45 min of venous occlusion. Cells a purple with trichrome staining with little to no evidence of RBC uptake. Degenerated tubular structure with cells with deep red pigment are common (arrow). All images taken at 100× magnification.

Injured tubular cells were often observed to contain red pigment and this was enhanced by venous clamping. Trichrome-stained sections revealed the presence of numerous red droplets within tubular cells following venous clamping without reperfusion (Figure 10A). This finding was similar to that observed following RBC trapping from arterial clamping with reperfusion. These red droplets were present in cortical tubules following both arterial and venous clamping without reperfusion; however, these droplets were much more prominent following venous clamping (Figure 10A, B, and D; *P* < 0.002). In trichrome-stained sections, following venous clamping without reperfusion, some cells appeared to turn bright red and were almost indistinguishable in color from the RBCs in the surrounding vasculature (Figure 10A). This reddening of the tubular cells was less prominent in the OM; however, injured, sloughed cells generally appeared a darker red (Figure 10E). This red color was absent in tubules of the inner stripe of the outer-medulla following arterial clamping (Figure 10C).

**Figure 11.**
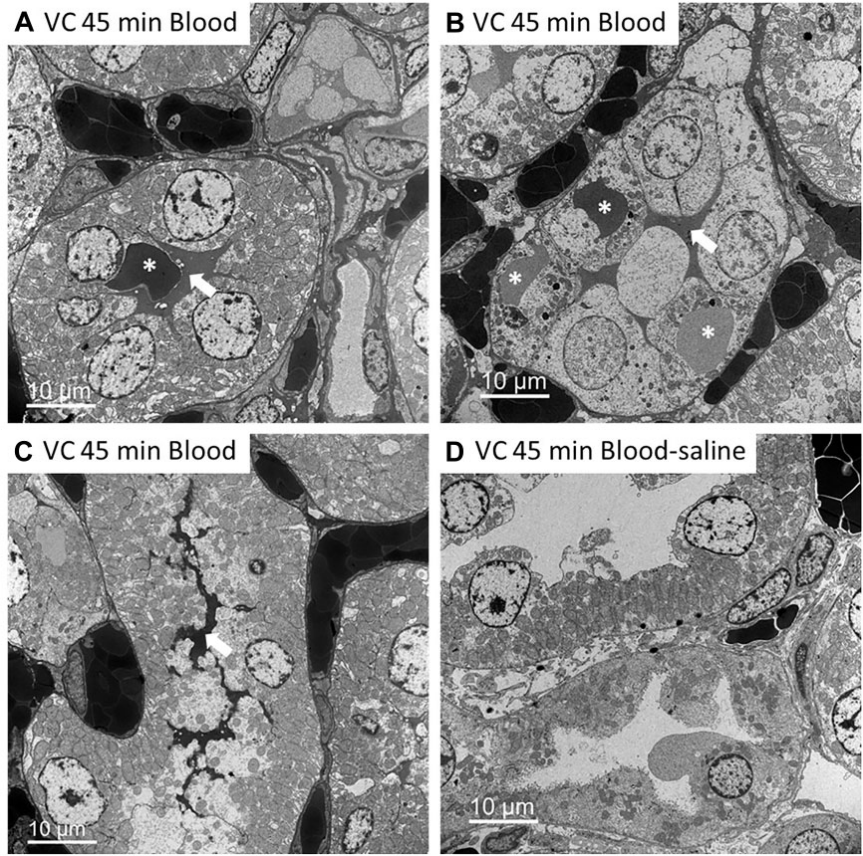
Venous clamping with and without blood: Electron-dense material is taken up and secreted/leaked into the tubular lumen. (Panels A, B, and C) are electron micrographs of the outer-medulla (OM) of a rat kidneys 45 min after venous clamping (VC) without reperfusion in blood-perfused kidneys. Asterisk (*) identifies electron-dense material within tubular cells that appears to be in different states of decay. Arrows point toward electron-dense material, which appears to be secreted into the tubular lumen. Electron-dense material can also be seen to fill the interstitial space. Suggesting these electron-dense structures are derived from RBCs. (Panel D), These electron-dense structures are absent and the lumen clear of electron-dense material in the OM of blood-free kidneys following the same 45 min of venous clamping. Some blood cells remain. The interstitial space is also clear of electron-dense material.

Transmission electron microscopy images from the OM of the kidneys of rats following 45 min of renal venous clamping without reperfusion confirmed electron-dense droplets within tubular cells (Figure 11A). These droplets often appeared to be in different states of decay, with varying electron density often observed (Figure 11B). Electron-dense material was also found within the basolateral invaginations of tubular cells and within the tubular lumen (Figure 11A–C). These electron-dense structures were completely absent from sections, and the lumen clear of electron-dense material, in blood-free kidneys following 45 min of venous clamping (Figure 11D).

**Figure 12.**
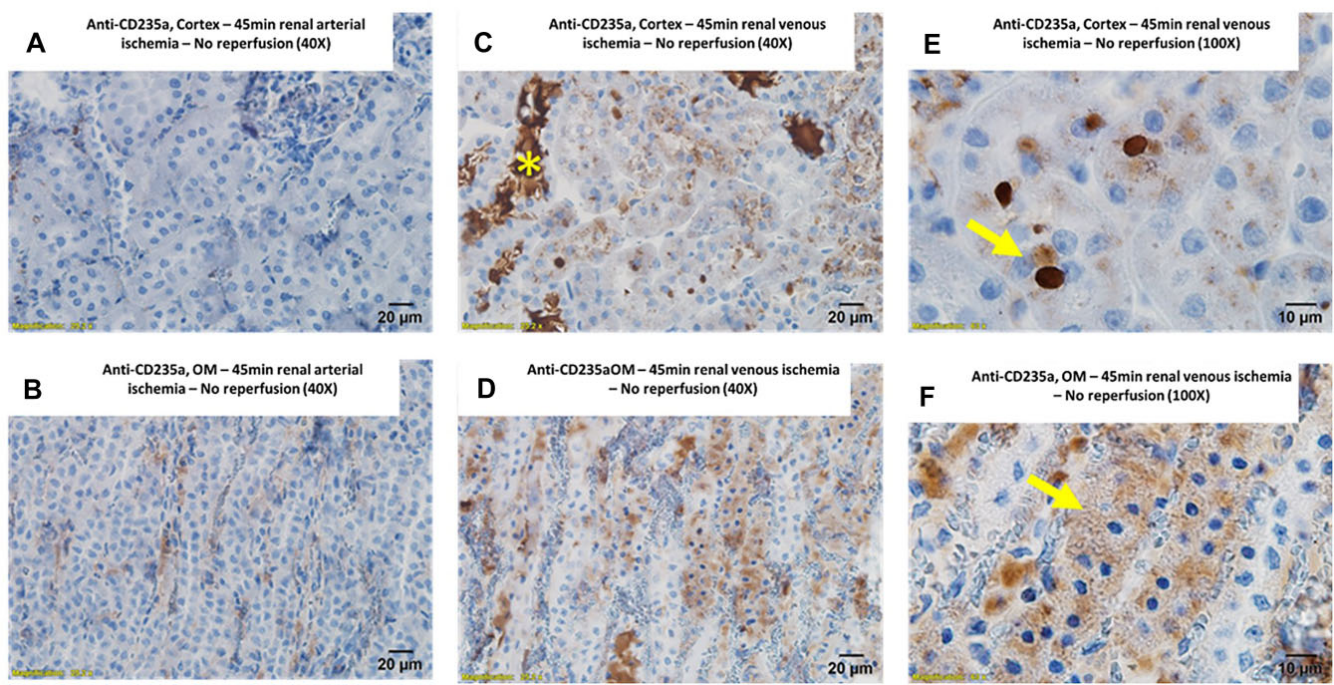
Venous clamping with blood: RBC membrane protein CD235a is concentrated in tubular casts and tubular cells following 45 min of venous clamping. (Panel A), CD235a staining in the cortex of a kidney following 45 min of ischemia from arterial clamping without reperfusion. Image is 40× magnification. CD235a staining (brown) is minimal and localized to within vascular structures. (Panel B), CD235a staining in the outer-medulla (OM) of a kidney following 45 min of ischemia from arterial clamping without reperfusion. Image is 40× magnification. CD235a staining is localized to within vascular structures. (Panel C), CD235a staining in the cortex of a kidney following 45 min of ischemia from venous clamping without reperfusion. Image is 40× magnification. Tubular casts stain strongly positive for CD235a. Most tubules also stain positive for CD235a. (Panel D), CD235a staining in the OM of a kidney following 45 min of ischemia from venous clamping without reperfusion. Image is 40× magnification. Tubular casts again stain strongly positive for CD235a with a diffuse staining pattern observed in many tubules. (Panel E), Higher magnification (100×) images of cortical tubules following 45 min of ischemia from venous clamping without reperfusion. Within cortical tubules, CD235a staining is observed within discrete droplets of various sizes. (Panel F), Higher magnification (100×) images of OM tubules following 45 min of ischemia from venous clamping without reperfusion. Within the OM, CD235a staining within tubules is diffuse and not within discrete droplets.

**Figure 13.**
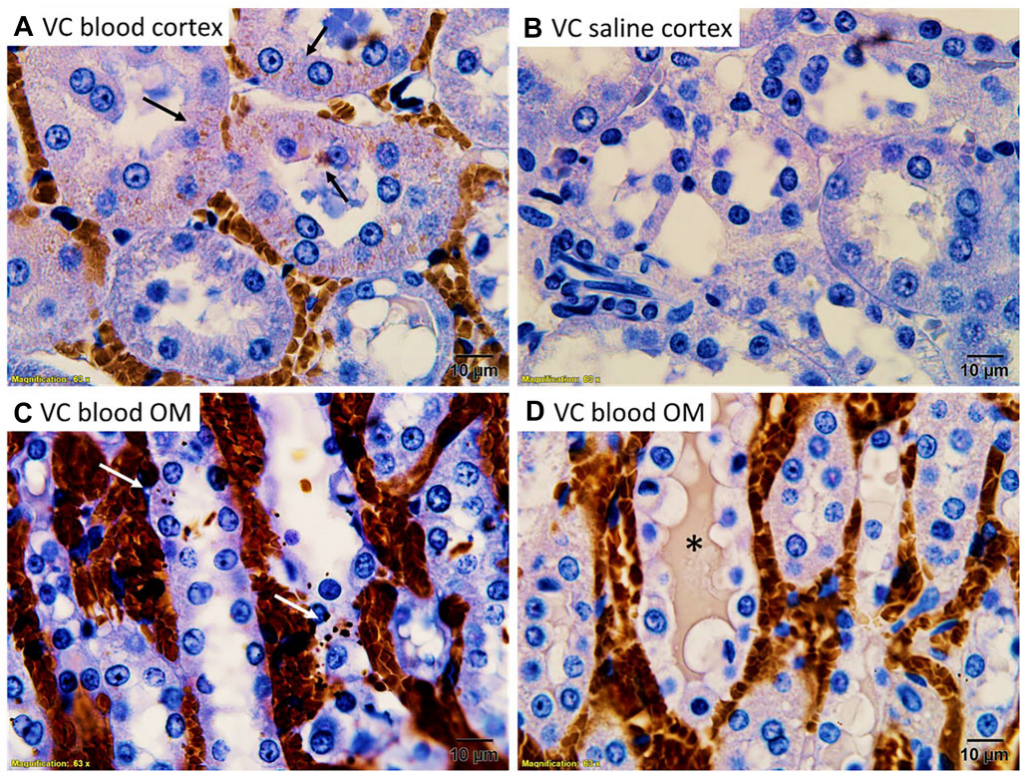
Venous clamping with and without blood: DAB staining for endogenous Hb peroxidase activity. Representative images of 3,3’-DAB (brown) staining for endogenous peroxidase activity from male WKY rats. (Panel A), Representative image of cortical tubules from rat blood-perfused kidney following 45 min of venous clamping (VC) with without reperfusion. Red blood cells stain positive due to the endogenous peroxidase activity of Hb. Absorption droplets (arrows) within the cytosol also stain positive for DAB. (Panel B), Representative image of cortical tubules from rat saline-perfused kidney following 45 min of venous clamping without reperfusion. Cortical tubules are negative for DAB-positive absorption droplets. (Panel C), Representative image of outer-medullary (OM) tubules from rat blood-perfused kidney following 45 min of venous clamping without reperfusion. The vessels are congested with tightly packed RBC that stain positive for DAB. Punctate areas of DAB staining can be observed within the tubules (arrows). (Panel D), Representative image of OM tubules from saline-perfused rat kidney following 45 min of venous clamping without reperfusion. The vessels are congested with tightly packed RBCs that stain positive for DAB. A DAB-positive tubular cast can be seen (*). All sections were stained together. Original magnification 100×.

Following venous clamping without reperfusion, many tubules in both the cortex and medulla had CD235a-positive droplets within their cytoplasm (Figure 12C and E). In the renal OM following ischemia from venous clamping, there was also diffuse CD235a staining within tubular segments (Figure 12D–F). Despite the same ischemic clamp time, tubular CD235a staining was minimal in both the cortex and medulla following ischemia from arterial clamping without reperfusion (Figure 12A and B). DAB staining of arterial and venous clamped kidneys without reperfusion followed a similar pattern to anti-CD235. Absorption droplets in cortical tubules from venous clamping, but not arterial clamped kidneys, stained positive for endogenous peroxidase activity with DAB (Figure 13). Similarly, tubular casts in the OM of venous clamp kidneys stained positive for endogenous peroxidase activity (Figure 13D). Staining of OM tubules following 45 min of venous clamping was observed as punctate areas of positive staining (Figure 13C).

**Figure 14.**
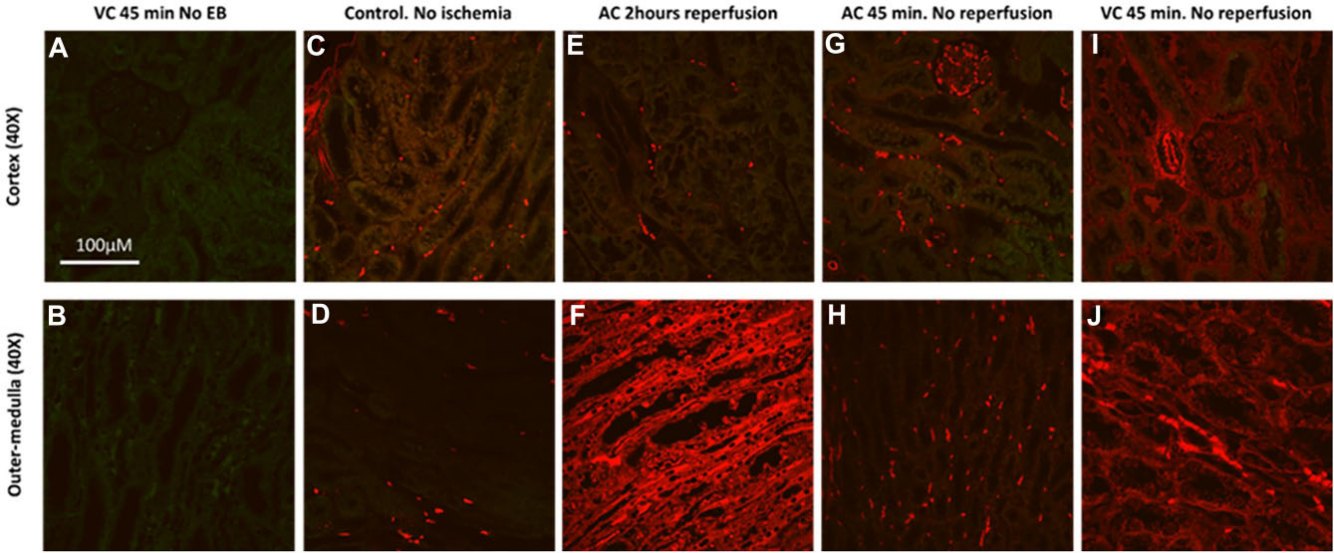
Control, arterial clamping with reperfusion and arterial and venous clamping without reperfusion: Comparison of EB fluorescence in cortex and outer-medulla. (Panel A), Representative image of kidney cortex from rat following 45 min of venous clamping (VC) without reperfusion that did not receive EB. No red fluorescence is present; however, green autofluorescence of the tubules is observed. (Panel B), Representative image of the kidney outer-medulla (OM) from rat following 45 min of VC without reperfusion that did not receive EB. No red fluorescence is present; however, green autofluorescence of the tubules is observed. Green autofluorescence is less than that of the cortical tubules. (Panel C), Representative image of kidney cortex from rat kidney without ischemic clamping (control), in which EB was circulated for 2 h and 45 min. Red blood cells and vessels are positive for EB red fluorescence. There is light positivity in the proximal tubules for EB. (Panel D), Representative image of kidney OM from rat kidney without ischemic clamping (control), in which EB was circulated for 2 h and 45 min. Red blood cells are positive for EB red fluorescence. The tubules are negative for EB. (Panel E), Representative image of kidney cortex from rat kidney following 2 h of reperfusion from 45 min of arterial clamp (AC) ischemia in which EB was administered. Red blood cells and vessels are positive for EB. The proximal tubules are negative. (Panel F), Representative image of kidney OM from rat kidney following 2 h of reperfusion from 45 min of arterial clamp kidney ischemia in which EB was administered. Almost all tubules and vascular structures are strongly positive for EB red fluorescence. (Panel G), Representative image of kidney cortex from rat kidney following 45 of arterial clamping without reperfusion (AC, no reperfusion), in which EB was administered prior to clamping. Red blood cells and vessels are positive for EB. The proximal tubules are mostly negative. (Panel H), Representative image of OM from rat kidney following 45 of arterial clamping without reperfusion (AC, no reperfusion), in which EB was administered prior to clamping. Red blood cells and vessels are positive for EB. The outer-medullary tubules are mostly negative. (Panel I), representative image of kidney cortex from rat kidney following 45 min of VC without reperfusion (VC, no reperfusion), in which EB was administered prior to clamping. Red blood cells and vessels are positive for EB. The proximal tubules are lightly positive for EB, indicated by red fluorescence. (Panel J), Representative image of OM from rat kidney following 45 of venous clamping without reperfusion (VC, no reperfusion), in which EB was administered prior to clamping. Red blood cells and vessels are positive for EB. The OM tubules are positive for EB, indicated by red fluorescence. Representative images from *n* = 3 kidneys in each group that were imaged. The original magnification of all images was 40×. Imaging settings (laser power/camera sensitivity) were the same for all images shown.

In order to confirm the uptake of extravasated blood proteins by both cortical and medullary tubules in areas of RBC trapping, we used confocal microscopy to image EB. In rats not injected with EB, both cortical and OM staining were negative (Figure 14A and B). In control rats injected with EB that did not undergo kidney ischemia reperfusion, RBCs and vascular structures stained strongly positive for EB but staining tubular structures in both the cortex and OM was low (Figure 14C and D). Following 2 h of reperfusion from 45 min of warm arterial clamping, tubular staining for EB in the cortical tubules was similar to that of non-ischemic controls (Figure 14E). In contrast, the OM tubules stained strongly positive for EB (Figure 14F). Red blood cell trapping does not occur following 45 min of arterial clamping without reperfusion. In these animals, both the cortical and OM tubules remained largely negative for EB (Figure 14G and H). Following 45 min of venous clamping without reperfusion, in which RBC trapping is present in both the cortex and OM, both the cortical and OM tubules stained positive for EB (Figure 14J). The intensity of staining, however, was less than that of the OM following 2 h of reperfusion from arterial clamping (Figure 14F).

**Figure 15.**
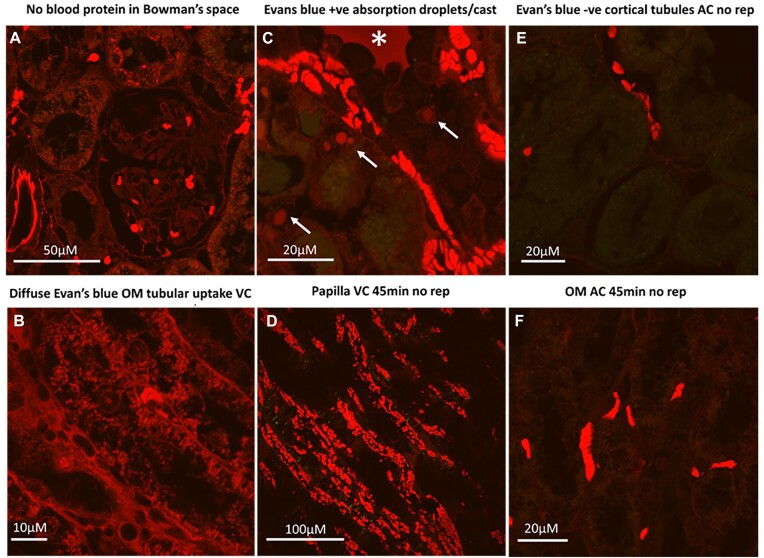
Arterial and venous clamping without reperfusion: Cellular and sub-cellular distribution of EB fluorescence. (Panel A), High-magnification representative image of kidney cortex from rat kidney following 45 of venous clamping (VC) without reperfusion in which EB was administered prior to clamping. Red blood cells and vessels are positive for EB. Evans blue-positive absorption droplets can be observed in some tubules, indicated by red fluorescence. There is no EB in the Bowman’s space of the glomerulus. (Panel B), High-magnification representative image of kidney outer-medulla (OM) from rat kidney following 45 of VC without reperfusion in which EB was administered prior to clamping. There is diffuse EB-positive red fluorescence within the tubules. (Panel C), High-magnification representative image of kidney cortex from rat kidney following 45 of VC without reperfusion in which EB was administered prior to clamping. Red blood cells and vessels are positive for EB. Evans blue-positive absorption droplets can be observed in some tubules, indicated by red fluorescence (arrows). Tubular casts are positive for EB (*). (Panel D), Representative image of kidney papilla from rat kidney following 45 of VC without reperfusion in which EB was administered prior to clamping. Only RBCs and vessels are positive for EB. (Panel E), High-magnification representative image of kidney cortex from rat kidney following 45 of arterial clamping (AC) without reperfusion in which EB was administered prior to clamping. Only RBCs and vessels are positive for EB. (Panel F), High-magnification representative image of kidney OM from rat kidney following 45 of AC without reperfusion in which EB was administered prior to clamping. Only RBCs and vessels are positive for EB. Representative images from *n* = 3 kidneys in each group that were imaged. Imaging settings (laser power/camera sensitivity) were the same for all images shown.

The cellular and sub-cellular distribution of EB was similar to that observed with anti-CD235a and DAB staining. Following venous clamping, large absorption droplets within the proximal tubules of the cortex were positive for EB (Figure 15A and C). Evans blue staining in the OM tubules was more diffuse (Figure 15B).

**Figure 16.**
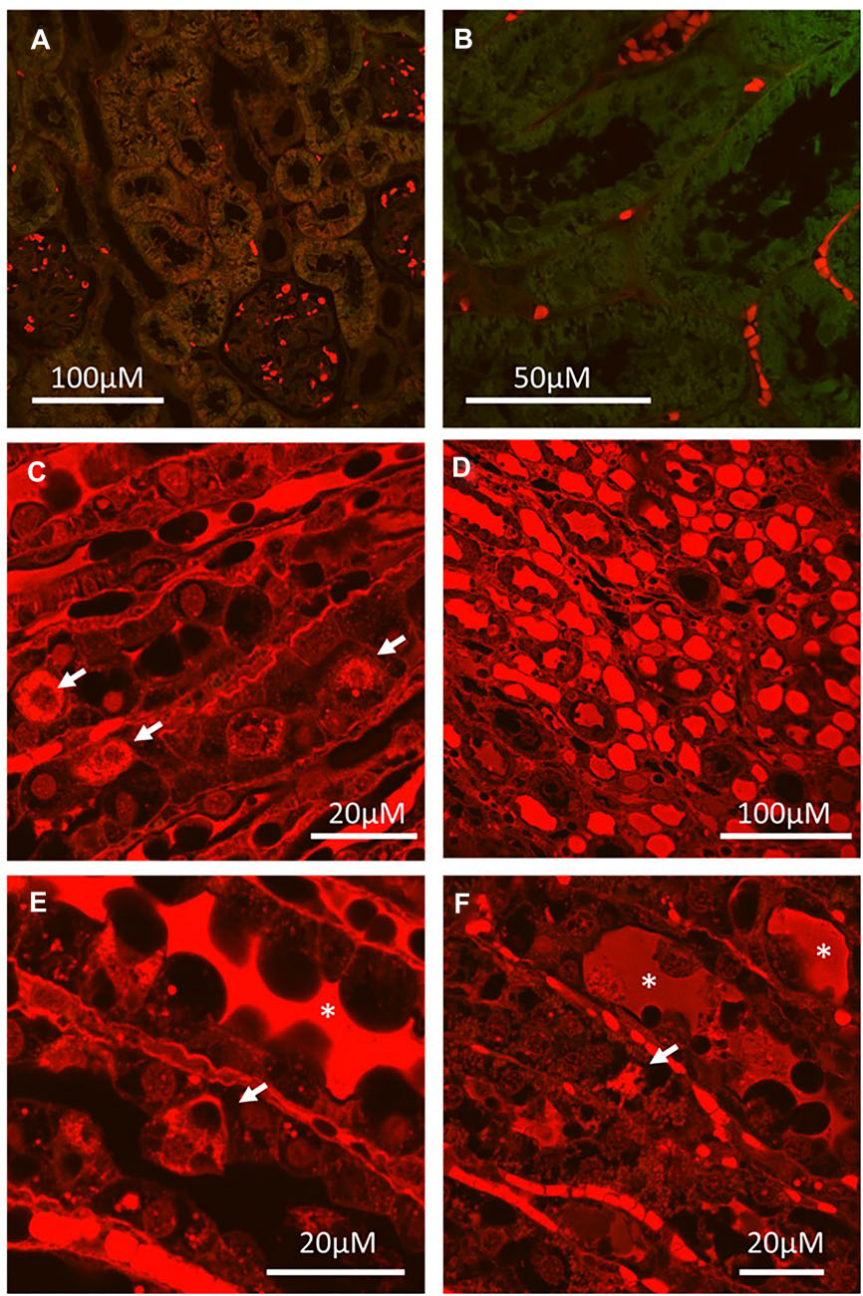
Arterial clamping with 2 h of reperfusion: EB localization. (Panel A and B), Representative images of kidney cortex from rat kidney following 45 min of arterial clamping (AC) with 2 h of reperfusion in which EB was administered. Red blood cells and vessels are positive for EB. Cortical tubules are mostly negative for red EB fluorescence. (Panel C), Representative image of high magnification of the kidney outer-medulla (OM) from rat kidney following 45 min of AC with 2 h of reperfusion in which EB was administered. There is diffuse EB-positive red fluorescence within the tubules. Some tubular cells appear bright red (arrows). (Panel D), Representative image of the kidney papilla from rat kidney following 45 min of AC with 2 h of reperfusion in which EB was administered. Many bright red tubular casts are present in the distal tubules. (Panel E), Representative image of high magnification of OM from rat kidney following 45 min of AC with 2 h of reperfusion in which EB was administered. A sloughing tubular cell is bright red (arrow). A tubular cast is also highly positive for red EB fluorescence (*). (Panel F), Representative image of high magnification of of OM from rat kidney following 45 min of AC with 2 h of reperfusion in which EB was administered. A sloughing tubular cell is bright red (arrow). Tubular casts are also strongly positive for EB (*). Representative images from *n* = 3 kidneys in each group that were imaged. Imaging settings (laser power/camera sensitivity) were the same for all images shown.

There was a close association between areas of tubular injury and blood protein uptake. Following 2 h of reperfusion from arterial clamping, injured tubular cells in the OM demonstrated marked uptake of EB, often staining as positive as RBCs (Figure 16C and E). Almost all tubular cells demonstrated red fluorescent droplets within their cytoplasm (Figure 16C). Similarly, OM tubular casts were strongly positive for EB (Figure 16E and F). Our data are consistent with extravasation of blood proteins from areas of RBC trapping as the source of tubular EB.

**Figure 17.**
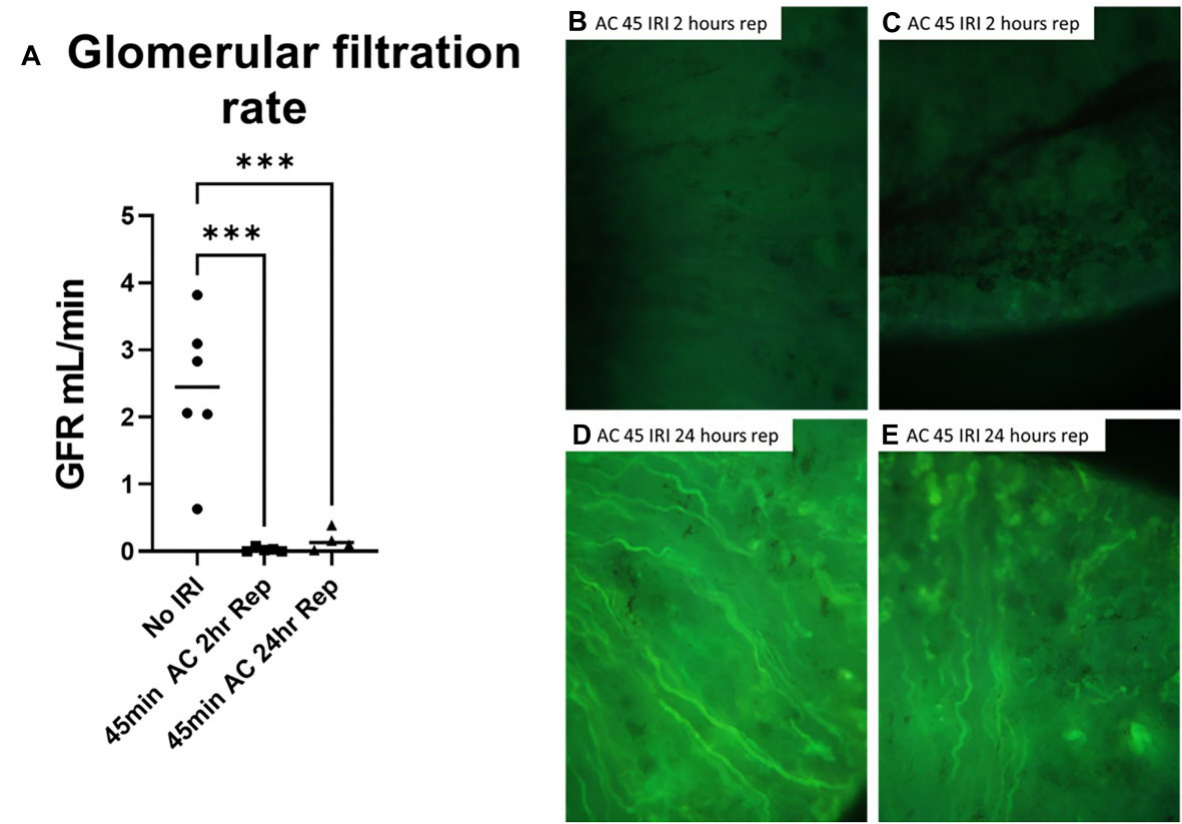
No ischemia-reperfusion control and arterial clamping with 2 and 24 h of reperfusion: GFR. (Panel A), Glomerular filtration rate (mL/min) measured by transcutaneous FITC-sinistrin clearance in male WKY rats. no ischemia controls (no IRI, circles, *n* = 6), 45 min bilateral warm arterial clamp (AC) ischemia with clearance measured between 0 and 2 h of reperfusion (squares, *n* = 5), and 45 min bilateral warm AC ischemia with clearance measured between 24 and 26 h of reperfusion (triangles, *n* = 4). Individual rats and mean shown. Statistics are the result of a one-way ANOVA with Tukey post-hoc test. (*** = *P* < 0.001). (Panels B and C), Representative images of the kidney cortex and outer-medulla (OM) from rats following 2 h of reperfusion from 45 min of warm bilateral ischemia from arterial clamping (5× original magnification). There was no evidence of filtration in kidneys between 0 and 2 h of reperfusion from arterial clamping. Neither the glomeruli nor tubules demonstrated the presence of FITC-sinistrin. (Panels D and E), Representative images of the kidney cortex and OM from rats following 26 h of reperfusion from 45 min of warm bilateral ischemia from arterial clamping. Some glomeruli and nephrons demonstrate the presence of FITC-sinistrin while others were negative.

There was no evidence of significant leakage of EB into the Bowman’s space of the kidneys following 2 h of reperfusion from arterial clamping (Figures 15A and 16A). In line with this, there was little evidence of significant EB uptake by cortical tubules following 2 h of reperfusion from 45 min of warm renal arterial clamping (Figure 16B). Further excluding filtration as a major source of EB uptake in the medullary tubules, we found that GFR was markedly reduced or completely absent in kidneys between 0 and 2 h of reperfusion from 45 min of warm bilateral arterial clamping when OM tubular EB uptake was observed (Figure 17A). We confirmed the absence of significant sinistrin clearance was due to loss of glomerular filtration rather than tubular back leak across this time period. Sinistrin fluorescence was most often completely absent in the glomeruli and tubules of thin sections of kidneys harvested 2 h post-reperfusion from 45 min of arterial clamping (Figure 17B and C). Only following 24 h of reperfusion was filtration in some glomeruli observed, although this remained patchy (Figure 17D and E).

**Figure 18.**
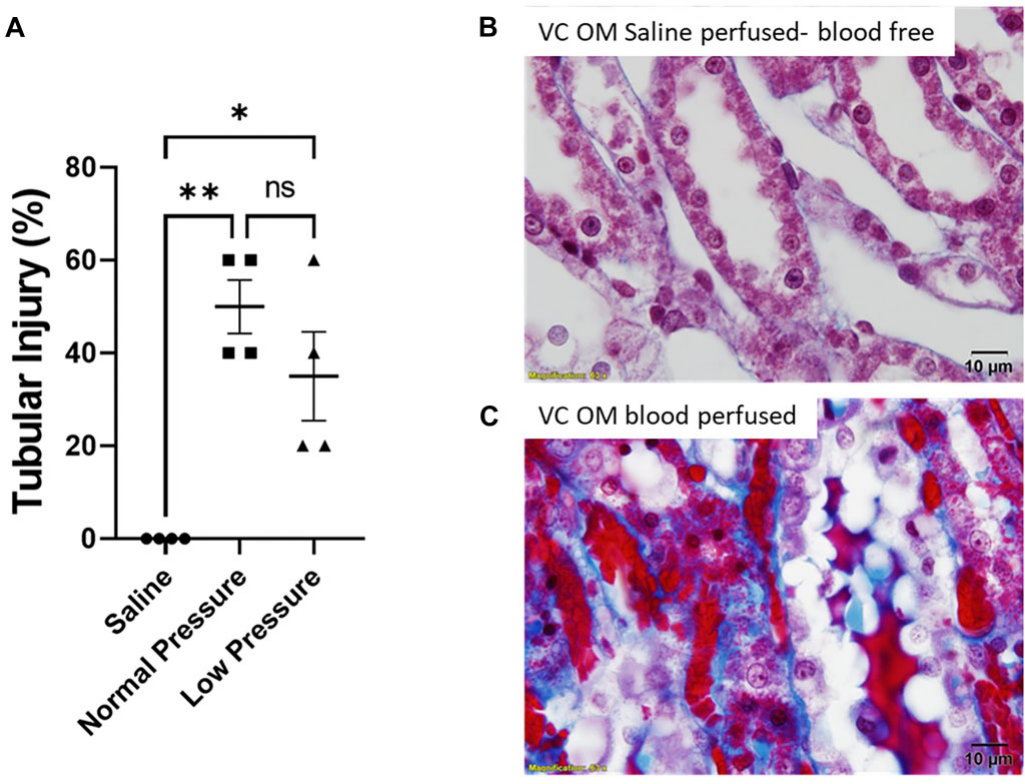
Venous clamping with and without blood: Effect of renal perfusion pressure on outer-medullary tubular injury in blood-free, saline-perfused and blood-perfused kidneys with venous clamping. (Panel A), Tubular injury scores of the outer-medulla (OM) following 45 min of renal venous clamping (VC) with no reperfusion in blood-free, saline-perfused (Saline, *n* = 4), normal renal perfusion pressure (normal pressure, *n* = 4), and low renal perfusion pressure (low pressure, *n* = 4) male Sprague–Dawley rats (11-13 wk of age). Tubular injury is reported as a score of 0-10 with cell swelling, tubular cast formation, and tubular injury scored. Values are expressed as mean ± SEM. Statistics represent a 2-way ANOVA with Sidaks multiple comparison test, *P < 0.05. Representative images of trichrome-stained sections of the OM following 45 min of venous occlusion with no reperfusion in saline-perfused “blood free” (Panel B) and blood-perfused (Panel C) kidneys are shown. Images taken at 100× magnification. Tubular casts, tubular degeneration, and cell swelling are prominent in the blood-perfused kidney but absent in saline-perfused kidneys following venous clamping.

We then tested the hypothesis that “tubular injury from venous clamping is mediated by blood.” Tubular injury, including OM tubular cast formation and cell swelling was almost exclusive to the blood-perfused kidney (Figure 18A–C; *P* < 0.0001). In kidneys that were perfused with saline to remove the blood prior to placing the venous clamp, the tubules were mostly uninjured (Figure 18A and B). Finally, we tested the hypothesis that reducing renal arterial perfusion pressure in the blood-perfused kidney would limit injury from venous clamping. Renal perfusion pressure was significantly lower in the low perfusion pressure group verses the normal perfusion pressure group being 38 ± 3 and 75 ± 2 mmHg, respectively (*P* = 0.0016). While tubular injury scores tended to be lower in the low renal perfusion pressure group compared to the normal renal perfusion pressure group, this did not reach statistical significance (Figure 18A, *P* = N.S).

## Discussion

The major finding of this study is that blood toxicity rather than warm ischemia time is likely to be responsible for much of the kidney injury observed in ischemic AKI. The localization of injury primarily to the outer medulla, along with evidence of a cortical-medullary oxygen gradient, led to the hypothesis that medullary hypoxia was largely responsible for kidney injury in AKI.^36^ Opposing this hypothesis, however, most evidence indicates that it is cortical rather than medullary perfusion that is reduced in human AKI.^37–40^ Trueta first demonstrated that a marked restriction or complete cessation of flow to the outer two-thirds of the kidney cortex may be associated with the maintenance of a normal or increased medullary circulation.^41^ This phenomenon has been documented in a variety of experimental situations^42,43^ and in kidney ischemia in men.^37–40^,^44^ This is consistent with the typical gross appearance of the AKI kidney, in which the cortex is pale and cortical vessels are collapsed, while the outer-medulla appears dark red and the outer-medullary vessels are dilated.^3,4,8,25^,^45–47^ While initially it was thought that the dusky red color of the outer-medulla represented medullary hyperemia, it has since become evident that the continued inflow of blood into the medulla during periods of kidney ischemia results in intense RBC congestion (RBC trapping) in the outer-medullary vasculature due to the failure of these medullary vessels to drain.^14,25^ Why then, if cortical ischemia is severe and the medullary circulation continues to be perfused (at least initially as RBC trapping develops), is ischemic/hypoxic kidney injury largely restricted to the OM? Ischemic/hypoxic injury also does not well explain the type of injury observed in human AKI. Ischemic injury is known to result in coagulative necrosis and the formation of ghost cells; however, such injury is rare in human AKI.^42^ Ischemic tubular injury also does little to explain the presence of heme casts in the distal nephron segments or the strong link between cell-free heme and ischemic AKI following shock.^45^,^48–51^ Our finding that extravasation of blood from the RBC-congested OM capillaries in AKI results in toxic injury to the tubular cells, likely provides an answer to these questions.

**Figure 19.**
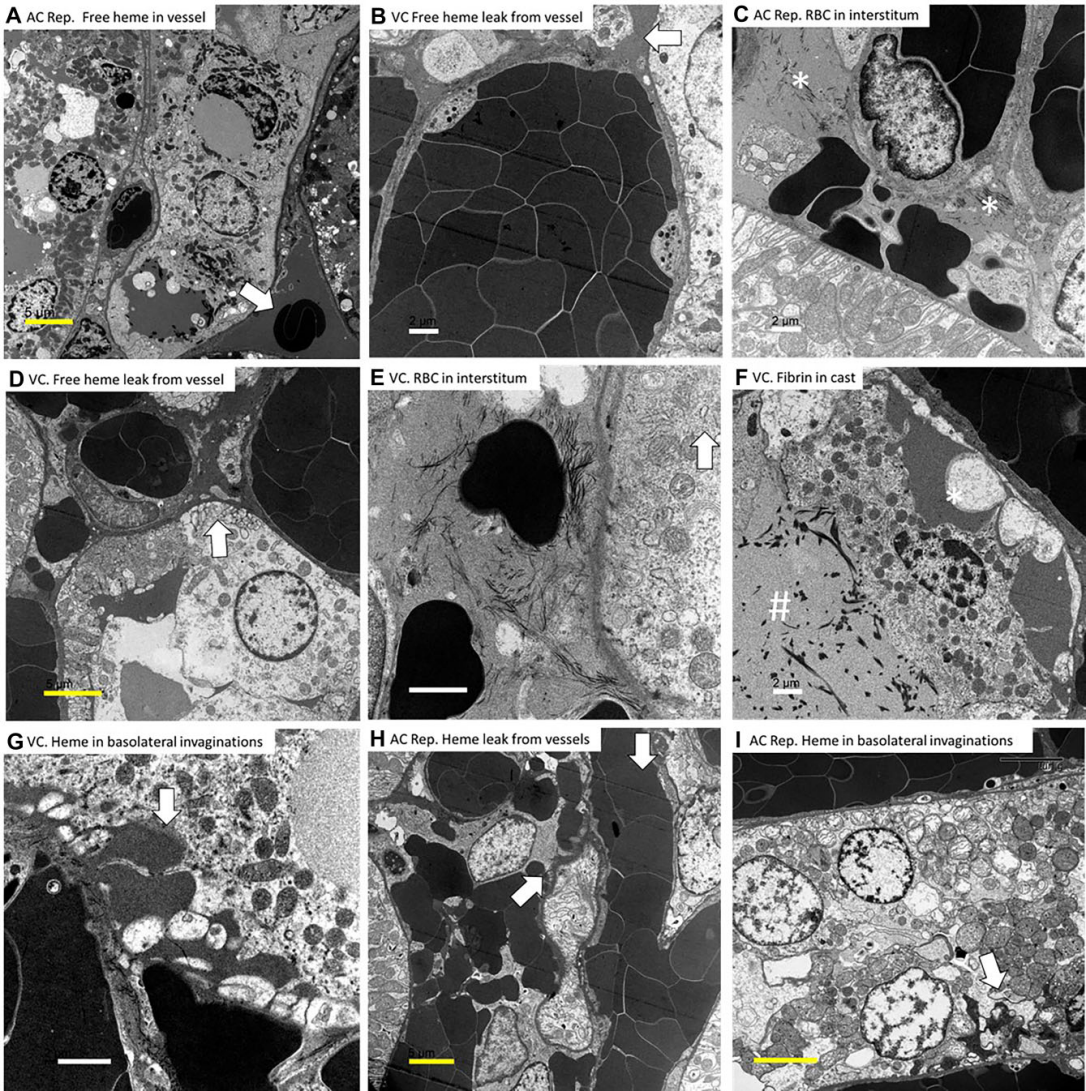
Evidence of tubular leakage of electron-dense material from congested microvessels following arterial clamping with reperfusion and venous clamping without reperfusion. (Panel A), Electron micrograph from rat kidney following 45 min of arterial clamping with 2 h of reperfusion (AC Rep), demonstrating electron-dense material (presumably Hb) filling the lumen of a capillary. Adjacent are injured tubular cells with electron-dense cytoplasm and absorption droplets. (Panel B), Electron micrograph from rat kidney following 45 min of venous clamping without reperfusion (VC), demonstrating electron-dense material (presumably Hb) filling the interstitial space around a capillary containing tightly packed RBCs with almost no plasma separating them. Note the vessel wall appears largely intact. (Panel C), Electron micrograph from rat kidney following 45 min of arterial clamping with 2 h of reperfusion (AC rep), demonstrating electron dense blobs (presumably from RBC) in interstitial space. Asterisk (*) denotes adjacent collagen fibers. (Panel D), Electron micrograph from rat kidney following 45 min of venous clamping (VC) without reperfusion, demonstrating electron-dense material filling the interstitial space surrounding RBC-congested capillaries. In places this material can been seen filling the basolateral invaginations of nearby tubular cells. (Panel E), Electron micrograph from rat kidney following 45 min of VC without reperfusion, demonstrating electron dense blobs (presumably from RBC) in the interstitial space. Asterisk (*) denotes adjacent collagen fibers. (Panel F), Electron micrograph from rat kidney following 45 min of VC without reperfusion, demonstrating electron-dense material in the space between a sloughing tubular cell and a congested capillary. Lower-density material fills the tubular lumen containing what looks like fibrin (#), suggesting a leak of plasma into the luminal space. (Panel G), Electron micrograph from rat kidney following 45 min of VC without reperfusion, demonstrating electron-dense material in the interstitial space between a RBC-congested vessel and tubular cell. Electron-dense material appears to fill and expand the basolateral invaginations of the tubule (arrow). (H) Electron micrograph from rat kidney following 45 min of AC with 2 h of reperfusion (AC Rep), demonstrating electron-dense material within the basolateral invaginations of the tubule (arrow). Similarly dense material fills the interstitial space between the tubular cell and a RBC-congested vessel. (Panel I), Electron micrograph from rat kidney following 45 min of arterial clamping with 2 h of reperfusion (AC Rep), demonstrating electron-dense material within the basolateral invaginations of the tubule (arrow). This was seen in tubular cells without any evidence of similar electron-dense material in the lumen or apical invaginations, suggesting the source was basolateral. Yellow scale bar = 5 µm. White scale bar = 2 µm.

Much evidence supports a role of heme toxicity in ischemic AKI.^4,45,48,50,52,53^ In fact, transient heme in the urine is such a prominent feature of ischemic AKI following shock in humans^54^ that the syndrome was once referred to as “hemoglobinuric nephrosis.”^4,45,55^ The source of these hemeproteins, however, has remained a mystery for over 70 yr.^45,56^ We hypothesized that extravasation of Hb or other blood proteins out of the RBC-congested OM capillaries may be the primary source of heme in AKI. We previously reported that RBC trapping is associated with marked tubular injury to the OM within 1-2 h of reperfusion from arterial clamping.^14^ As such, in the current study, we first examined rat kidneys following 2 h of reperfusion from 45 min of ischemia from warm bilateral arterial clamping. At this time point, we found evidence of extravasation of blood, including RBCs and their contents from congested vessels and uptake by nearby tubular cells. Evidence supporting the extravasation of blood proteins and RBC material includes: (1) transmission electron micrographs demonstrating the presence of electron-dense material (presumably free Hb from damaged RBC) within and surrounding RBC-congested vessels (Figure 19), and (2) the presence of blood proteins (EB) and Hb peroxidase activity (DAB) outside the vascular space in areas where RBC trapping was present. As we and others have found that the vasculature endothelium remains largely intact at this time,^13,14^ we speculate that the extravasation of this fluid is likely due to increased intravascular pressures associated with RBC trapping and venous obstruction (Figure 19). Our data suggests this extravasated material is taken up nearby kidney tubules, a process that may explain toxic tubular injury in this setting. Evidence for the uptake of this extravasated material by the tubular cells includes: (1) electron microscopy images demonstrating that there is electron-dense material surrounding the RBC congested vasculature and that this was often continuous with similar electron-dense material filling the basolateral invaginations of tubular cells (Figure 19), (2) electron-dense material within absorption droplets within tubular cells, which was restricted to areas of RBC trapping and stained positive for CD235a, Hb, DAB, and EB. While we were unable to confirm the specificity of the anti-CD235a antibody we utilized for RBCs, CD235a staining was restricted to areas of RBC trapping. The cellular and sub-cellular distribution of CD235a staining was also similar to our other blood markers. Further supporting the extravasation of blood proteins into the tubular lumen, following both venous clamping and arterial clamping with reperfusion, what looked like fibrin deposits were also often present in the tubular lumen (Figure 19F). Taken together, our data are highly consistent with the uptake of extravasated blood and RBC material by the tubules in areas where RBC trapping occurs.

While the source of tubular heme and protein casts in AKI has traditionally been thought to be through filtration, our data are not consistent with this. Both the Evans-blue albumin complex and Hb are large molecules that are not generally filtered. While it is possible that glomerular injury following ischemia results in increased permeability to large molecules, we found no evidence of significant EB filtration within 2 h of reperfusion when OM tubular uptake was occurring. In rats 2 h following reperfusion from 45 min of arterial clamping, both the Bowman’s capsule and proximal tubules were negative for EB staining (Figure 16). Furthermore, our data using trans-cutaneous sinistrin clearance indicate that there is little or no filtration of even freely filtered molecules in the early hours of reperfusion (Figure 17). Despite this, the distal and the OM tubules stain strongly positive for EB. Bright red fluorescent casts fill the many distal tubules and injured tubular cells stain almost as positive as blood. Extravasation of blood from the OM vessels has previously been reported following AKI.^24^ Extravasation of blood from areas of RBC trapping not only explains the localization of this material to RBC-congested areas in the absence of filtration but also the almost complete absence of plasma in much of the congested vasculature (Figure 19B–D).

To determine whether tubular injury was directly due to blood toxicity or was secondary to prolonged ischemia, we compared renal arterial and venous clamping without reperfusion. OM RBC congestion is minimal during the clamp period with arterial clamping. Upon reperfusion from arterial clamping, RBC congestion forms due to obstruction of the venous vessels that drain the medulla.^14,22,23^ Using renal venous clamping, we were able to mimic this venous obstruction during the clamp period, promoting early RBC congestion prior to reperfusion. We found that despite the same 45-min warm ischemia time, tubular injury was much more prominent, and tubular cast formation exclusive to venous clamping when compared with arterial clamping without reperfusion. Tubular injury with 45 min of venous clamping, although milder (presumably due to reduced time of exposure to RBC congestion), resembled that observed at 2 h post-reperfusion from arterial clamping, with tubular cells demonstrating evidence of heme uptake and toxic tubular injury. This injury could not have been due to increased intra-renal pressures alone. Confirming the role of blood toward injury, this type of injury was absent in saline-perfused kidneys following venous clamping. Together, our data indicate that extravasation of blood and blood toxicity, rather than extension of ischemia time, is responsible for the early devastating tubular injury occurring in RBC-congested areas of the kidney OM following reperfusion from arterial clamping in the rat IRI model.^14^ While extension of ischemic time to the OM does not appear to be responsible for early injury to the OM, our data do not exclude a role of RBC trapping and delayed reperfusion to promote further injury or delayed recovery of the medullary tubules at later time points.

Red blood cell congestion and tubular RBC toxicity likely explain differences in kidney injury with venous compared with arterial clamping. Studies from rodent models have shown that short periods of warm venous clamping are more detrimental than warm arterial clamping, but that this is reversed with longer ischemia times.^57–61^ As with venous clamping, congestion forms during the ischemic period, even with short periods of venous clamping, toxic tubular injury develops. Our observations with venous clamping indicate that RBC congestion often resolves within minutes of releasing the venous clamp. As RBC congestion often rapidly resolves following removal of the venous clamp, the time in which tubular RBC toxicity occurs with venous clamping is limited to the clamp period. In contrast, while OM vascular congestion commonly develops following reperfusion from warm arterial clamping, this response is highly variable, particularly with shorter ischemic periods.^1,14^ When congestion does develop, however, it is prolonged, lasting 24-48 h.^2^ As such, when the ischemic period is short, vascular congestion from arterial clamping is unlikely to develop, resulting in reduced toxic kidney injury compared with similar lengths of venous clamping. When the ischemic period is prolonged, RBC congestion is more likely to develop, resulting in an overall much longer period of congestion and greater tubular toxicity than that of venous clamping. While we did observe RBC trapping in both the cortex and OM following venous clamping, and this was associated with proximal tubular injury, in humans, RBC trapping only rarely occurs in the kidney cortex, such as following renal venous thrombosis. As such, the focus of our studies is on RBC trapping and injury in the OM, which commonly occurs after severe renal ischemia or shock.^25^

**Figure 20.**
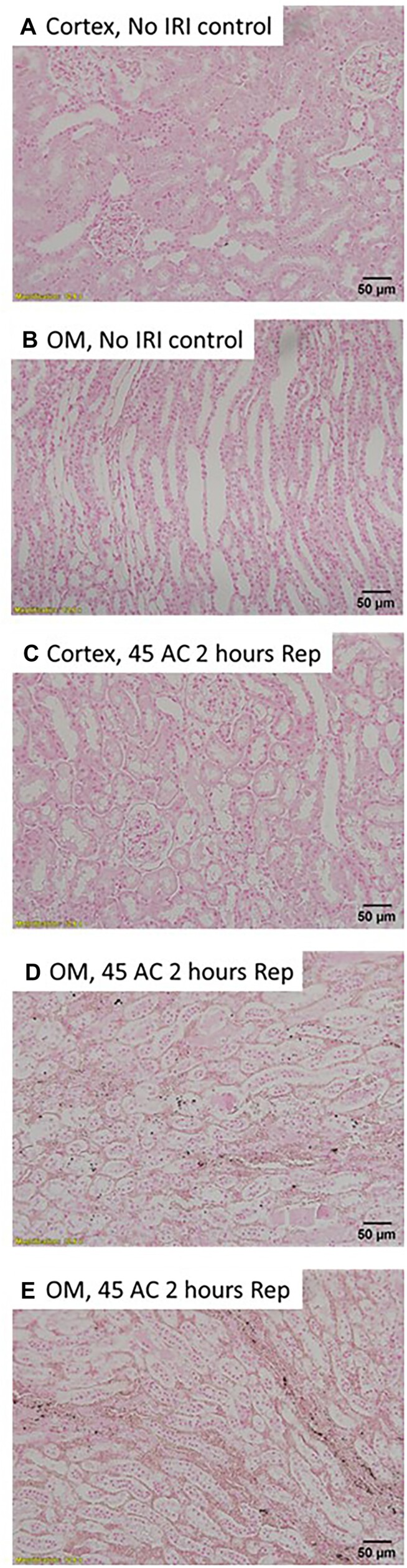
Arterial clamping with reperfusion and no ischemia control kidneys: Prussian blue staining for non-heme bound iron. Representative images of Prussian blue staining staining for non-heme bound iron from male WKY rats. (Panel A), Representative image of cortex from control kidney that did not undergo ischemia reperfusion (no IRI control). No iron deposits are observed. (Panel B), Representative image of the outer-medulla (OM) from control kidney that did not undergo ischemia reperfusion (no IRI control). No iron deposits are observed. (Panel C), Representative image of the cortex from kidney that was subject to 45 min of warm arterial clamp ischemia with 2 h of reperfusion (45 AC 2 h Rep). No iron deposits are observed. (Panel D), Representative image of the OM from kidney that was subject to 45 min of warm arterial clamp ischemia with 2 h of reperfusion (45 AC 2 h Rep). Iron deposits are observed both in vascular and tubular structures (dark specs). (Panel E), Representative image of the OM from kidney that was subject to 45 min of warm arterial clamp ischemia with 2 h of reperfusion (45 AC 2 h Rep). Image demonstrates localization of iron deposits (dark specs) to RBC congested VR bundles. This is consistent with RBC and Hb breakdown and the release of Hb-free iron from RBC.

While our data suggest that RBC trapping-associated tubular injury occurs secondary to the tubular uptake of extravasated blood proteins, the blood protein(s) responsible for injury remain unclear. A likely source of tubular injury may be free Hb, which can cause cellular iron accumulation and ferroptosis of tubular cells.^62,63^ Free Hb, released from damaged RBCs, has been reported to enter both proximal tubular cells^64^ and tubules in the distal nephron between the membrane invaginations on their surface.^34^ When the transport capacity of the cell is overwhelmed, this Hb then accumulates in electron-dense droplets within the cell, similar to those observed in our study.^64^ In our study, we observed evidence of electron-dense material filling the basolateral invaginations of tubular cells, suggesting free Hb from RBCs degenerating in the congested vasculature is being absorbed. Consistent with the release and breakdown of Hb from RBCs, we also observed evidence of free iron accumulation in the OM. This was most prominent around congested VR, indicating RBC breakdown as the likely source (Figure 20). Interestingly, high circulating levels of free Hb produce tubular injury remarkably similar to that observed in the congested renal OM. This includes the formation of heme-positive casts in the distal nephron,^19,34,45,48,54^ cell sloughing,^34,65^ darkened cell cytosol,^34^,^65–67^ cytoplasmic vacuolization,^34,68^ and the invasion of mononuclear cells into the vasa-recta.^19,34,45,69^ Critically, our new data indicate that the RBC congested medullary vasculature may be the primary source of this toxic heme in ischemic AKI. Further studies are needed to determine whether RBC trapping-associated injury is primarily mediated by Hb and/or other blood proteins.

Our data indicate that RBC trapping occurs and causes toxic tubular injury even at low renal perfusion pressures. Venous clamping, blockage, or physiological blockage of the vessels that drain the medulla following renal arterial ischemia, results in an increase in intravascular pressure within the kidney.^70,71^ To examine the role of renal perfusion pressure on RBC trapping and injury, we compared blood-perfused kidneys with venous clamping at normal and low renal perfusion pressures. As RBC trapping and tubular injury were similar at both normal and low renal perfusion pressures, this suggests that RBC trapping and tubular blood toxicity may occur even at low arterial pressures, such as in shock. Our data do not exclude a role of high renal perfusion pressures in worsening toxic tubular injury from RBC trapping. Wei *et al*. have reported that high renal perfusion pressure results in worsening renal injury with venous clamping.^72^ Trapped RBCs are so tightly packed together that they form polygonal shapes.^2,22,23^ It is possible that higher renal perfusion pressures, by increasing both the physical compression of the RBC membrane and subsequent RBC lysis and the rate of fluid extravasation of this fluid, may worsen tubular toxicity when there is RBC trapping.

Our finding that blood is extravasated from areas of RBC trapping leading to toxic tubular injury can explain much of the pathology observed following ischemic AKI in humans. The clinical syndrome and histopathology of AKI are remarkably similar across AKI of diverse etiology.^45^,^73–75^ Histopathological injury observed in AKI from various pathologies incudes lipid degeneration or vacuolization of the thick ascending limb of the Loop of Henle,^2,17,18^ large numbers heme casts in the distal nephrons, degeneration and desquamation of the lower nephrons, and rupture and non-occlusive thrombosis of the thin-walled veins and VR.^2,3,17,21^ Remarkably, the renal cortex most often appears largely uninjured.^2,19,21,23^ In line with our previous study,^14^ we found severe tubular injury in the OM within 2 h of reperfusion from arterial clamping. Severe tubular sloughing in the OM has often been viewed as post-mortem autolysis; however, evidence suggests this may be incorrect. Lerrolle *et al*. found that RBC trapping along with cytoplasmic degeneration and detachment of the tubular cells from their basement membrane were present in kidney biopsies from humans with AKI taken immediately following death, where autolysis would not yet have occurred.^76^ Our data indicate that the rapid sloughing of the medullary tubules upon reperfusion of the kidney^14^ is secondary to toxic injury from extravasation of blood from the RBC congested medullary vasculature. The timing of this, early in reperfusion, corresponds with the period in which there is transient excretion of Hb in the urine early in the course of the development of oliguria in patients with ischemic AKI.^4^ Remarkably, in rats, many of these severely injured OM tubules appear to regenerate by 24 h of reperfusion.^14^ While we speculate that similar regeneration of the tubular epithelium may be responsible for obscuring the most severe injury from RBC trapping in humans, our data suggests that many of the key pathological features of ischemic AKI found in humans at later time points can likely be attributed RBC trapping and extravasation of blood proteins from the OM capillaries. A number of studies have reported electron-dense droplets and heme-positive luminal casts in the distal nephron segments following reperfusion from ischemia.^4,42,45,65,68^,^77–79^ The assumption has been that this material entered the tubular lumen by filtration and that electron-dense droplets in distal nephron segments represent absorption droplets or degenerate organelles.^77–79^ Opposing this assumption and consistent with our observations in rats, studies of human kidneys following AKI demonstrate little evidence that blood or heme proteins traversed the lumen of the upstream nephron segments.^48,54,56^ Further, heme casts are present in the distal nephron following ischemic AKI, even in cases in which a source of filtered heme, such as hemolysis or crush injury, is absent.^45,66^ Our data provides explanation to these seemingly disparate findings. That is, rather than being filtered, RBC material in the lumen of distal tubular cells was extrasavated from the RBC congested medullary capillary circulation before being taken up by tubular cells and secreted or leaked into the tubular lumen. This mechanism would explain the presence of heme casts in the lumen of uninjured tubules as well as evidence that hemoglobinuria absent shock/kidney ischemia, such as paroxysmal nocturnal hemoglobinuria, does not cause renal failure.^48,49,56^ That is, the luminal heme itself is not highly toxic, but rather represents the product of a process that overwhelmed many tubular cells (cellular uptake and secretion of extravasated RBC material across the basolateral membrane). A number of authors have also noted the proximity of tubular injury to microvessels.^4,68^ This relationship, where injury is greatest in tubules with close proximity to vessels, is consistent with extravasation of toxins from the vasculature and toxic injury to nearby tubular cells, with cells more distant to the extravasated capillary material being spared. Finally, muddy brown urinary casts are pathognomonic of tubular injury in ischemic AKI. These casts are known to contain heme; however, the origin of this heme is unknown.^80^ Our findings suggest that these muddy brown heme casts are likely to be made up of sloughed, degenerated cells that took on heme from RBC material extravasated from the congested medullary vasculature.

Red blood cell trapping has traditionally been thought to promote tubular injury by extending ischemia time to the OM. Our data contest this assumption, demonstrating that RBC trapping in the kidney OM vasculature results in rapid extravasation and uptake of blood material by tubular cells, resulting in toxic injury to the tubules early in the reperfusion period. Further, our study identifies RBC trapping and extravasation of blood from the OM as the source of distal tubular heme casts in ischemic AKI, the source of which has remained a mystery for over 70 yr.^4,48,56^ Blood toxicity from RBC trapping appears to be a major component of tubular injury in ischemic AKI, which has not previously been recognized. As injury from blood toxicity closely mimics that observed in human kidneys following ischemic AKI, this suggests blood toxicity may be the primary cause of outer-medullary tubular injury in ischemic AKI. If confirmed, this would explain the failure of interventions targeting medullary hypoxia to improve outcomes in ischemic AKI^81–83^ and open the door for new approaches targeting RBC trapping and tubular blood toxicity to prevent nephron loss in ischemic AKI.

## Disclosures

None.

## Supplementary Material

zqad050_Supplemental_File

## Data Availability

The data underlying this article are available in “Figshare” digital Repository at DOI: 10.6084/m9.figshare.24045147

## Notes

Translational statement: Our study challenges the concept that tubular injury in the outer-medulla in ischemic acute kidney injury is primarily mediated by hypoxia. Instead, our data indicate that tubular injury maybe largely due to extravasation of blood from the microvasculature and blood toxicity. We find that following periods of ischemia, trapping of red blood cells in the medullary capillaries results in extravasation of components of blood into the tubular interstitium. We demonstrate that this leads to tubular heme cast formation and toxic tubular injury, independent of warm ischemia time. Our data explain much of the pathology observed following ischemic acute kidney injury in humans, including, the location of injury to tubules in the outer-medulla directly adjacent to thin-walled veins/capillaries, distal tubular heme casts and the “heme” content of “muddy brown” urinary casts.
