# Supplementary material for: Extravasation of Blood and Blood Toxicity Drives Tubular Injury from RBC Trapping in Ischemic AKI
Source: Function (Oxf). 2023 Sep 4;4(6):zqad050. doi: 10.1093/function/zqad050 (PMC10519276; doi:10.1093/function/zqad050)
Supplement: zqad050_Supplemental_File [file zqad050_supplemental_file.docx]

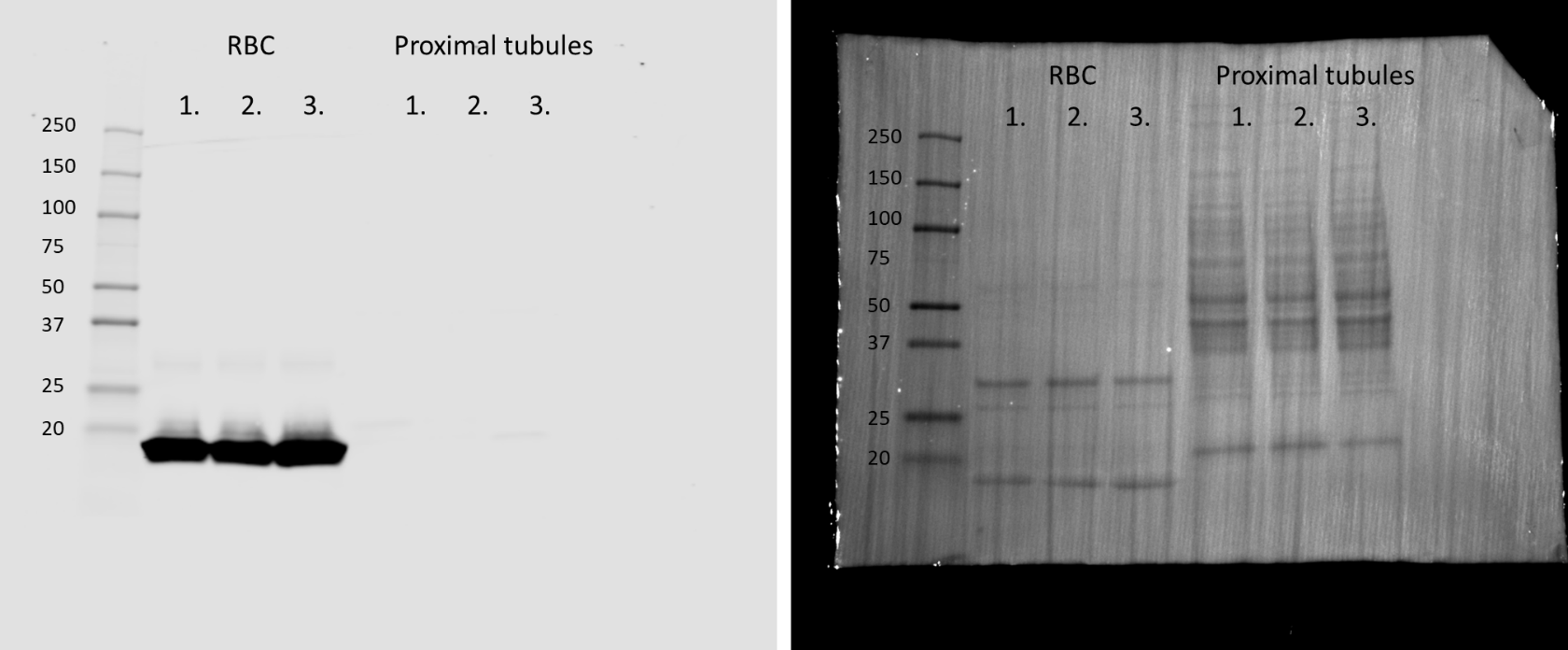


**Supplementary Figure 1. Western blot anti-hemoglobin.** **Left panel)** Western blot. 30µg of RBC and proximal tubular cytosolic protein was loaded per lane for Hemoglobin antibody**.** Each lane (1-3) represents an individual animal. RBC, red blood cell membrane protein. Proximal tubules, membrane protein isolated from proximal tubular isolation. There is a single band corresponding to the molecular weight of hemoglobin only in the lanes loaded with red blood cell membrane protein. **Right panel**) Ponceau Staining. As RBC did not show B-actin, we use Ponceau Staining as our loading control.


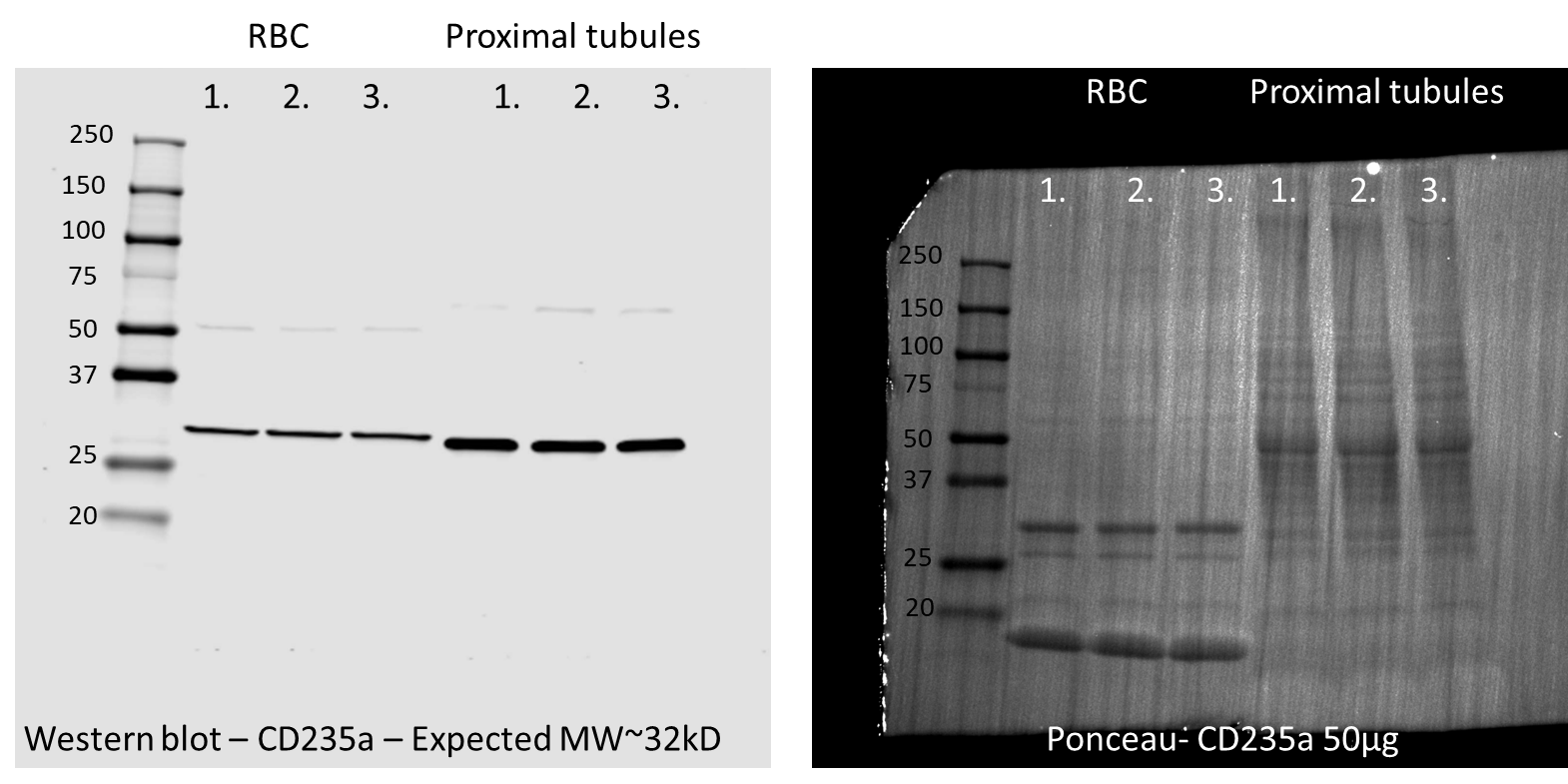


**Supplementary Figure 2. Western blot anti-CD235a.** **Left panel) Left panel)** Western blot. 50µg of RBC and proximal tubular cytosolic protein was loaded per lane for CD235a antibody**.** Each lane (1-3) represents an individual animal. RBC, red blood cell membrane protein. Proximal tubules, membrane protein isolated from proximal tubular isolation. There is a single band corresponding to the molecular weight of CD235a in RBC membrane fraction. A second non-specific band with a slightly lower molecular weight is observed in the proximal tubular membrane fraction. **Right panel**) Ponceau Staining. As RBC did not show B-actin, we use Ponceau Staining as our loading control.
